# Overlapping expression and co-operative function of the zebrafish *pcdh15* paralogs

**DOI:** 10.1038/s42003-026-10289-7

**Published:** 2026-05-18

**Authors:** Paul W. Chrystal, Lisa Kuzmanova, Jingpin Liu, Alexa Izvorean, Ishmael Majeed, Jefferson F. Tjoenardi, Qian Lin, Vincent Tropepe

**Affiliations:** Department of Cell & Systems Biology, University of Toronto, Toronto, ON Canada

**Keywords:** Retinal diseases, Mechanisms of disease, Neurodegeneration, Disease model, Cytoskeleton

## Abstract

With orthologs for >70% of human genes, zebrafish represent a popular model for investigating development and disease, particularly when murine models are unsuitable. One caveat of zebrafish genetic models is the prevalence of paralogs, where compensation and transcriptional adaptation can complicate our understanding. Usher syndrome type 1 F (USH1F), caused by *PCDH15* mutations, exemplifies the need for alternatives models; the primary site of injury, calyceal processes (CP), are uniquely lacking from photoreceptors of nocturnal rodents. Here we demonstrate that contrary to previous reports, zebrafish *pcdh15a/b* paralogs are not absolutely restricted to ear and eye tissues respectively. Instead, both paralogs are expressed and required in the mechanosensitive hair cells and retinal photoreceptors. Double mutants present the most severe phenotypes: loss of kinocilial and stereocilial links in the ear, and disorganization of CPs, inner/outer segment detachment, and photoreceptor cell death. We also demonstrate that ear and eye-specific phenotypes may be isolated via isoform-specific knockout. These data highlight the strengths of zebrafish to study pathomechanism in vivo. They should also caution researchers from defining paralogs as discrete, absolutely restricted functional units without exceptional evidence. When possible, double mutants may be preferable for studying gene function, and modelling diseases where humans have a single ortholog.

## Abstract (web summary)

Contrary to previous reports, zebrafish paralogs *pcdh15a* /*15b* both contribute to ear and eye development but display isoform expression bias. Double mutants have the most severe phenotypes, including retinal cell death consistent with USH1F patients.

## Introduction

Since the 1980s, zebrafish (*Danio rerio*) have become one of the most utilized model organisms for biological research^1,2^. As a basal vertebrate with a body plan that closely aligns with *Homo sapiens*, there are many advantages to using zebrafish; low cost of husbandry, rapid, *ex utero* development, transparency during embryogenesis, and an extensive genetic toolbox combine for a tractable, in vivo model for dissecting gene function. The publication of the zebrafish reference genome over a decade ago revealed that >70% of human genes have at least one fish ortholog^3^. Subsequently, zebrafish have proven invaluable models for understanding development and disease.

A distinctive feature of zebrafish as a genetic model is the abundance of duplicate genes (paralogs). Beyond the two rounds of whole genome duplication (WGD) common to all vertebrates (2 R hypothesis)^4,5^, zebrafish underwent an additional, teleost-specific WGD since their radiation from tetrapods^6,7^. Under relaxed selection pressure, most duplicates were rapidly lost^8,9^, however >20% of human orthologs have two zebrafish paralogs^3,10^. These duplicates persist through mechanisms of 1) neofunctionalization – addition of novel functions distinct from the ancestral gene, and 2) subfunctionalization – discrete parsing of ancestral function between the two paralogs. In zebrafish, subfunctionalization often manifests as expression pattern divergence^11–13^, where two paralogs possess a more restricted expression domain than their ancestral ortholog, ensuring that both copies must be retained to fulfil the full complement of functions^14^. However, the retention of paralogous genes has significant implications for disease modelling in zebrafish. For example, introduction of a frameshift mutation can trigger nonsense-mediated decay of the mRNA and upregulation of related (adapting) genes in a process known as transcriptional adaptation (TA)^15,16^. Upregulation of paralogous genes can compensate for gene loss, leading to a milder phenotype, and possibly masking true gene function^17,18^.

**Fig. 1:**
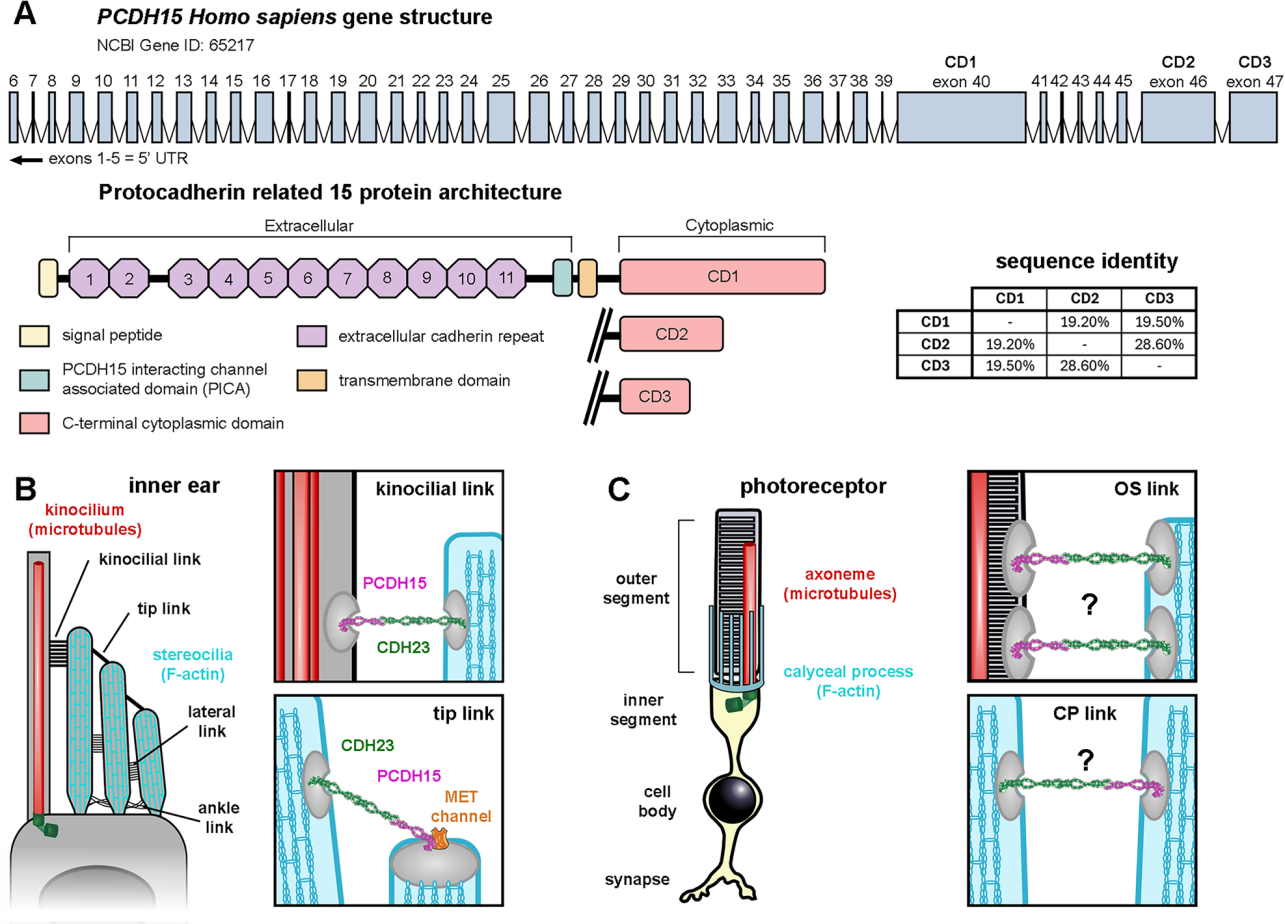
The role of protocadherin related 15. **A** *PCDH15* is a 1.8 Mb gene that encodes the atypical cadherin member protocadherin related 15. This protein has three major categories of isoform defined by the inclusion of exon 40, 46, or 47. The resulting proteins have a common domain architecture in the extracellular aspect, but differ in their C-terminal cytoplasmic domain (CD1, CD2, CD3). The three C-terminal domains have low sequence homology, suggestive of distinctive roles and binding partners. **B** Within hair cells of the inner ear, protocadherin related 15 plays a role in cell adhesion and audition. Localized to the transient kinocilium, protocadherin related 15 homodimers bind cadherin-related 23 (*CDH23*) homodimers, forming the kinocilial link, critical for planar polarity of the tissue. Protocadherin related 15 also localizes to the tips of stereocilia and forms the tip link to a neighbouring stereocilia. Deflection of stereocilia causes bending of the tip link and influx of Ca^2+^ and K^+^ through mechanoelectrical transduction (MET) channels. **C** Retinal function of protocadherin related 15 is incompletely understood but *PCDH15* is expressed in photoreceptors and localizes to the proximal outer segment. Actin filopodia (calyceal processes) arising from the inner segment and surrounding the base of the outer segment overlap with protocadherin related 15 localization. There has been speculation about protocadherin related 15 forming molecular links between 1) the CPs and OS (OS link) homologous to kinocilial links and/or 2) links between adjacent CPs (CP links) analogous to tip links.

One example of a human gene that is represented by two zebrafish paralogs is *PCDH15*, encoding the cell adhesion protein protocadherin related 15. Human *PCDH15* encodes 22 alternative transcripts (Table S1)^19^ that vary in length, exon usage and termination codon. With the exception of one variant, all *PCDH15* isoforms share a common architecture: a signal peptide, 11 extracellular cadherin repeats, PICA domain, transmembrane domain, and a C-terminal domain (Fig. 1A). Isoform sequence diversity is largely attributed to the alternative usage of three large 3’ exons. The resulting products, categorized as PCDH15-CD1, -CD2, or -CD3 (C-terminal cytoplasmic domain 1-3)^20^, differ in their binding capacities, localization patterns, and redundancies^21–23^. Both zebrafish paralogs (*pcdh15a* and *pcdh15b)* have retained this domain architecture, and both genes possess the alternate C-terminal domains: -CD1, CD2 and -CD3 (Table S2). However, previous studies suggested strict subfunctionalization: *pcdh15a* is expressed in inner ear hair cells and lateral line neuromasts, whereas *pcdh15b* is exclusive to the retinal photoreceptors^24^. This division has enabled separate modelling of auditory and visual phenotypes associated with the human disease by mutating only a single paralog^24–26^.

Disease variants in human *PCDH15* cause Usher syndrome type 1 F (USH1F), an autosomal recessive condition characterized by congenital sensorineural hearing loss, vestibular disturbance, and progressive rod-cone dystrophy^27,28^. The pathomechanism of disease is closely aligned to the function of protocadherin related 15 within cells of the inner ear and retina (Fig. 1B, C). In mechanosensitive hair cells of the inner ear, homodimeric protocadherin related 15 binds homodimeric cadherin-related 23 (*CDH23*) between adjacent stereocilia (actin filopodia) and kinocilia (microtubule-based cilia). These molecular handshake interactions provide adhesion at kinocilial links and contribute to mechanoelectrical transduction (MET) at tip links via gating of MET ion channel activation (Fig. 1B)^29,30^. In the retina, protocadherin related 15 localizes to the base of the photoreceptor outer segment (OS). This region of the cell is characterized by actin processes (calyceal processes) that bridge the inner segment/outer segment boundary and form a collar around the basal OS^31–33^. It has been theorized that protocadherin related 15 may play a structural role in the highly specialised photoreceptor cells by forming molecular bridges analogous to the tip links or kinocilial links of the inner ear (Fig. 1C)^33,34^. However, the retinal role of *PCDH15* remains poorly understood. While murine mutants have been invaluable for uncovering the auditory roles of Usher proteins, their utility for retinal function is limited; mice lack definitive calyceal processes, and *Pcdh15* mutants fail to replicate the retinal dystrophy of USH1F patients^33,35,36^. These limitations underscore the need for alternative vertebrate models.

Here, we use zebrafish *pcdh15* single and double mutant models to investigate the function of protocadherin related 15 in mechanosensation and vision. We demonstrate that the existing dichotomic expression pattern model for *pcdh15a/b* is an oversimplification. Instead, both paralogs contribute during development of the sense organs with variable phenotypes attributable to each gene separately; double mutants almost always displaying a more severe phenotype. The retinal phenotype is particularly illustrative of paralog cooperativity since *pcdh15b* single mutants have mild phenotypes even in aged animals, but severe retinal cell dysmorphology and cell death when both paralogs are mutated. These studies highlight the potential for genetic redundancy in zebrafish with paralogous genes and emphasise the need for double mutants when studying human recessive disease. Whether by functional compensation or transcriptional adaptation, paralogous zebrafish genes can mask the functional consequences of gene loss if only single copies are mutated.

Superseding paralog-specific expression, we uncover an isoform-specific bias for *pcdh15* in the sense organs. The retina predominantly expresses *pcdh15*-CD1, whereas the inner ear has a strong *pcdh15*-CD3 bias. Targeted mutagenesis of the -CD1 or -CD3 isoforms separately isolates the severe photoreceptor and sensory hair cell phenotypes in a tissue-specific manner. These data refine our understanding of protocadherin related 15 biology and suggest that therapeutic strategies for USH1F should account for isoform diversity.

## Results

### Adult *pcdh15*^−/−^ homozygotes display mild outer segment dysmorphology without cell death

The *pcdh15b*^uot15^ allele was reported in 2021 and carries a 17 bp insertion within the 5^th^ coding exon of zebrafish *pcdh15b*. This mutation leads to a frameshift, and introduction of a premature termination codon that affects all isoforms^26^. Although larval *pcdh15b*^−/−^ homozygotes exhibit early photoreceptor dysmorphology and ribbon synapse defects, they die by 20 days post fertilization (dpf) with no evidence of retinal cell death. Since USH1F patient retinal dystrophy is progressive, we wanted to determine whether aged *pcdh15b*^−/−^ homozygotes showed more severe retinal phenotypes.

To increase survival of the homozygotes, a more permissive husbandry protocol was developed. The genotype was identified at 3 dpf via larval tail biopsy^37^, and homozygotes were raised together at low density, preventing outcompeting for food. Under these conditions, ~20% *pcdh15b*^−/−^ homozygotes reached adulthood (Figure S1A). We speculate that the low survival rate may reflect the failure to inflate the swim bladder of most homozygous larvae (Figure S1B, C), a common finding in mutant lines with deficiency in equilibrioception^38^. The *pcdh15b*^−/−^homozygotes had a transient reduction in globe size at 2 dpf which resolved at later timepoints, but were otherwise indistinguishable from WT controls during development (Figure S1D).

**Fig. 2:**
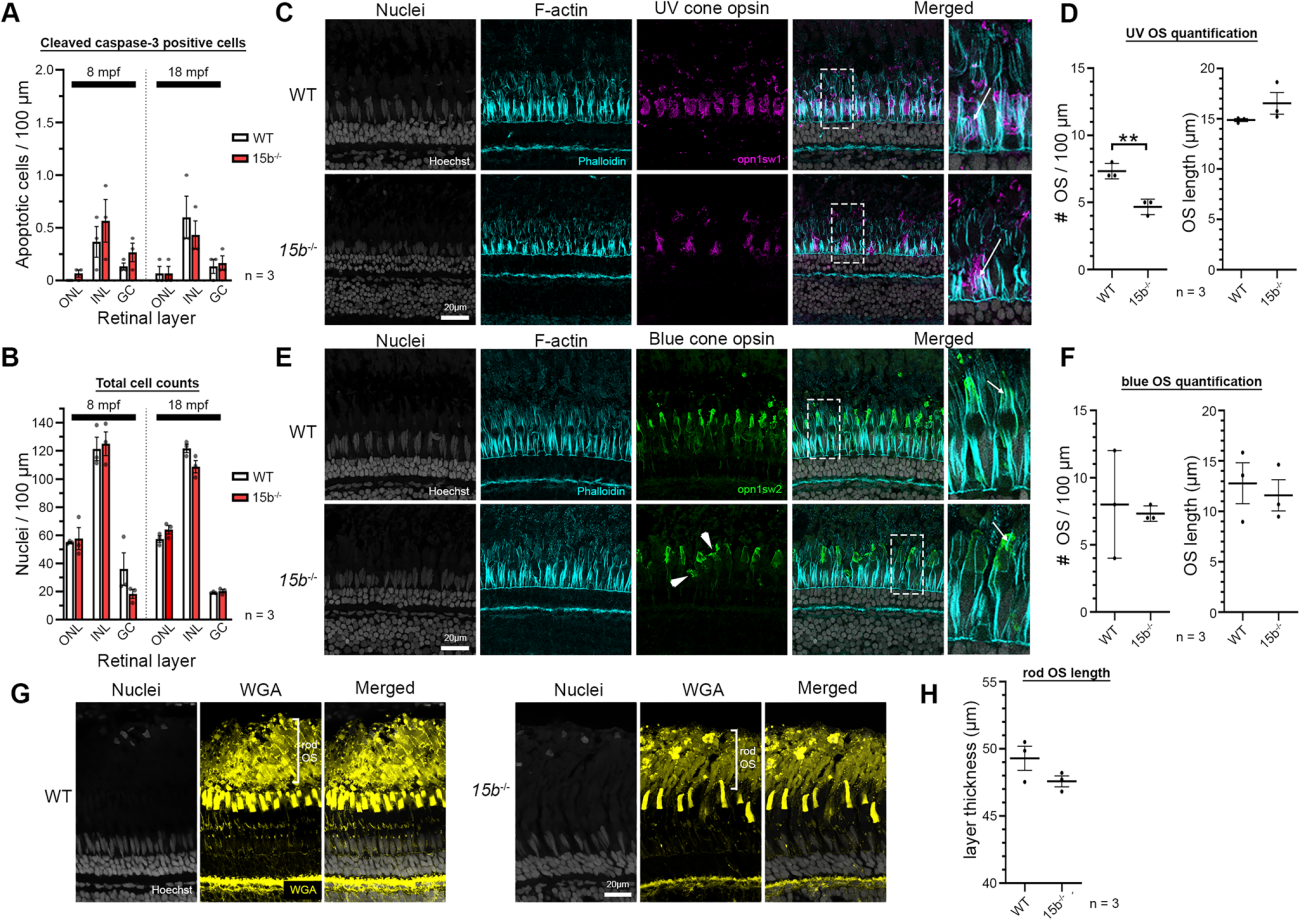
*pcdh15b*^−/−^ homozygotes develop mild retinal dysmorphism but not dystrophy. **A** *pcdh15b*^−/−^ homozygotes do not have an increased rate of apoptosis in any retinal layer at 8 mpf or 18 mpf. **B** Absolute counts of the nuclei in each retinal layer also show no change between WT and *pcdh15b*^−/−^ homozygotes at either timepoint. **C** Representative confocal micrographs of the 18 mpf retina stained for UV cone opsin and F-actin. In *pcdh15b*^−/−^ homozygotes the UV cone OS is dysmorphic, but actin processes remain closely associated (arrow). **D** Upon quantification, UV cone OS abundance was found to be reduced in *pcdh15b*^−/−^ homozygotes, but the length of OSs was unchanged (*P* = 0.0048; *P* = 0.2583, respectively; Welch’s *t*-test). **E**) Representative confocal micrographs of the 18 mpf retina stained for blue cone opsin and F-actin. There is some evidence of dysmorphic or mislocalized blue cone OSs (arrowheads), but actin processes were present (arrow). **F** There was no difference in blue cone OS abundance or length in *pcdh15b*^−/−^ homozygotes (*P* = 0.8010; *P* = 0.6693, respectively; Welch’s *t*-test). **G** WGA stains rod OS (bracket) and a subset of cone OSs in the 18 mpf retina. **H** There was no significant difference in thickness of the rod OS layer between WT and *pcdh15b*^−/−^ homozygotes (*P* = 0.1875; Welch’s *t*-test).

The *pcdh15b*^−/−^ homozygotes and WT siblings were raised to 8 and 18 months post fertilization (mpf) under standard rearing conditions (14:10 300 lux light:dark cycles) prior to sacrifice. Despite the advanced age, the mutant retina showed no increase in apoptosis at either time point studied (Fig. 2A). Furthermore, quantification of nuclei number in each retinal layer determined that there was no difference in cell number between the *pcdh15b*^−/−^ homozygotes and age-matched WT controls (Fig. 2B). However, we did observe mild changes to retinal histology. At 18 mpf the UV cone outer segments (OSs) were dysmorphic with reduced abundance in the central retina. These changes were not associated with any differences in UV cone OS length (Fig. 2C, D), and calyceal processes remained adjacent to the OS (Fig. 2C arrows). Blue cone OSs also appeared morphologically abnormal, with occasional OS detachment from the rest of the cell (Fig. 2E arrowhead). However, abundance and length of blue cone OSs were unchanged (Fig. 2F).

Since USH1F causes a human rod-cone dystrophy we also probed *pcdh15b*^−/−^ homozygotes with fluorescently conjugated wheat germ agglutinin (WGA) to stain the rod outer segments^39,40^. In adult zebrafish retinas we found that WGA strongly stained the rod OS layer and a subset of cone OSs (Fig. 2G). The high density of rod OSs prevented individual cells from being measured, but the thickness of the rod OS layer was unchanged in *pcdh15b*^−/−^ homozygotes. In combination, these data demonstrate that the *pcdh15b*^−/−^ adults develop mild outer segment dysmorphism, but do not develop severe retinal dystrophy like that observed in USH1F patients.

### The *pcdh15* paralogs have overlapping expression in the eye and ear with isoform-specific bias

**Fig. 3:**
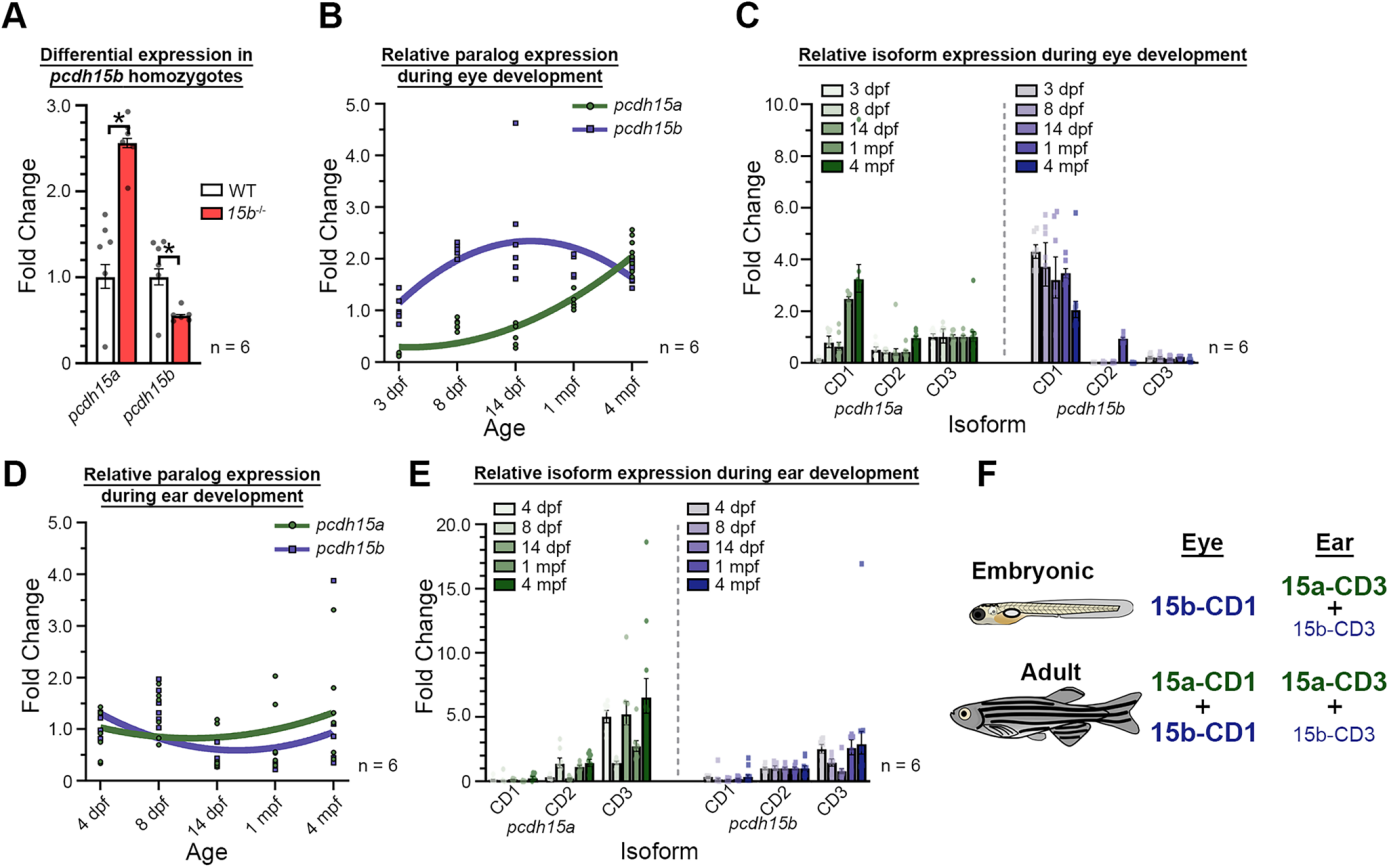
Zebrafish *pcdh15* genes display isoform-specific biases in the eye versus the ear. **A** Relative ocular expression levels of *pcdh15a* are increased, while *pcdh15b* levels are decreased, in *pcdh15b*^−/−^ homozygotes (*P* = 0.0213, *P* = 0.0315, unpaired *T*-test). **B** Ocular expression of *pcdh15a* and *pcdh15b* over time relative to 3 dpf *pcdh15b* levels. **C** Ocular expression of the c-terminal isoforms of *pcdh15a* and *pcdh15b* over time (relative to the *pcdh15a* CD3 transcript) demonstrates that the CD1 isoform is the major contributor from both paralogs. **D** Otic expression of *pcdh15a* and *pcdh15b* over time relative to 4 dpf *pcdh15a*. **E** Otic expression of the c-terminal isoforms of *pcdh15a* and *pcdh15b* over time (relative to the *pcdh15b* CD2 transcript) demonstrates that the CD3 isoform is the major contributor from both paralogs. **F** Revised model for the expression of *pcdh15* in the zebrafish sense organs. During early development, *pcdh15b*-CD1 is the major contributor to ocular tissues, but -CD1 isoforms from both paralogs contribute during adulthood. In the embryonic and adult otic tissues, CD3 isoforms from both paralogs are expressed, but *pcdh15a* expression is typically greater.

Given that the *pcdh15b*^uot15^ allele introduces a premature termination codon, we hypothesized that transcriptional adaptation (TA) may explain the mild retinal phenotype. Upregulation of adapting genes is a common occurrence when a truncated mRNA undergoes nonsense-mediated decay^18^. In ocular tissues of the *pcdh15b* mutant, we observed a significant reduction in *pcdh15b* mRNA abundance coincident with an ~2.5x increase in *pcdh15a* abundance (Fig. 3A). These data are consistent with TA and provide a potential explanation for the mild retinal phenotype. However, we were surprised to observe that in WT ocular tissue, there was only a three-fold difference in baseline expression of *pcdh15a* and *pcdh15b* at 8 dpf (mean ΔCq value 15a: 4.6, 15b: 3.0, Figure S2A). This challenges previous reports where little or no expression of *pcdh15a* was reported in the eye beyond 1 day post fertilization^24^, whereas isoform-specific analyses did not assess retinal expression^25^.

To test whether both *pcdh15* paralogs were expressed in the eye, we therefore designed a set of three, non-overlapping, isoform-agnostic PCR primers (Figure S2B) and performed endpoint PCR on cDNA samples generated from eye dissections. Expression at three timepoints that span larval to adult stages revealed that both paralogs were indeed expressed in the eyes (Figure S2C). We therefore sought to more fully characterise the paralog-specific expression pattern of *pcdh15* in the developing sense organs. We also investigated the isoform-specific expression levels using primers targeted to the three major isoform classes: -CD1-3.

In cDNA derived from ocular tissue, we found that *pcdh15b* had greater relative expression at early developmental timepoints, but expression of *pcdh15b* remained relatively stable ( ~ 2-fold increase between larval and adult tissues). Comparatively, expression of *pcdh15a* was low at early developmental timepoints but rose rapidly ( > 14-fold increase). By adulthood, there was a comparable contribution from both paralogs in the eye (Fig. 3B). Using isoform-specific primers (-CD1, -CD2 and -CD3), we found that the most abundant isoform of both paralogs in the eye was the -CD1 isoform (Fig. 3C). Using this same approach, we then assessed expression from cDNA generated from dissected auditory organs. Expression of both paralogs remained relatively stable during the developmental time course but *pcdh15a* expression was higher at later stages (Fig. 3D). Assessment of individual isoforms revealed that the -CD3 isoform of each paralog were typically the most abundant (Fig. 3E). While we did observe expression from the -CD2 transcripts of both genes, these isoforms were never the major contributors to ocular or otic tissues. In combination, these data suggest that instead of paralog-specific roles for *pcdh15a/b* in the zebrafish sense organs, there exists an isoform-specific bias in expression between ocular and otic tissues: the -CD1 isoform is preferentially expressed in the developing eye, whereas the -CD3 isoform is more abundant in the ear (Fig. 3F). Critically, this transcriptional bias is consistent between paralogs.

### Generating a *pcdh15a*; *pcdh15b* double mutant

**Fig. 4:**
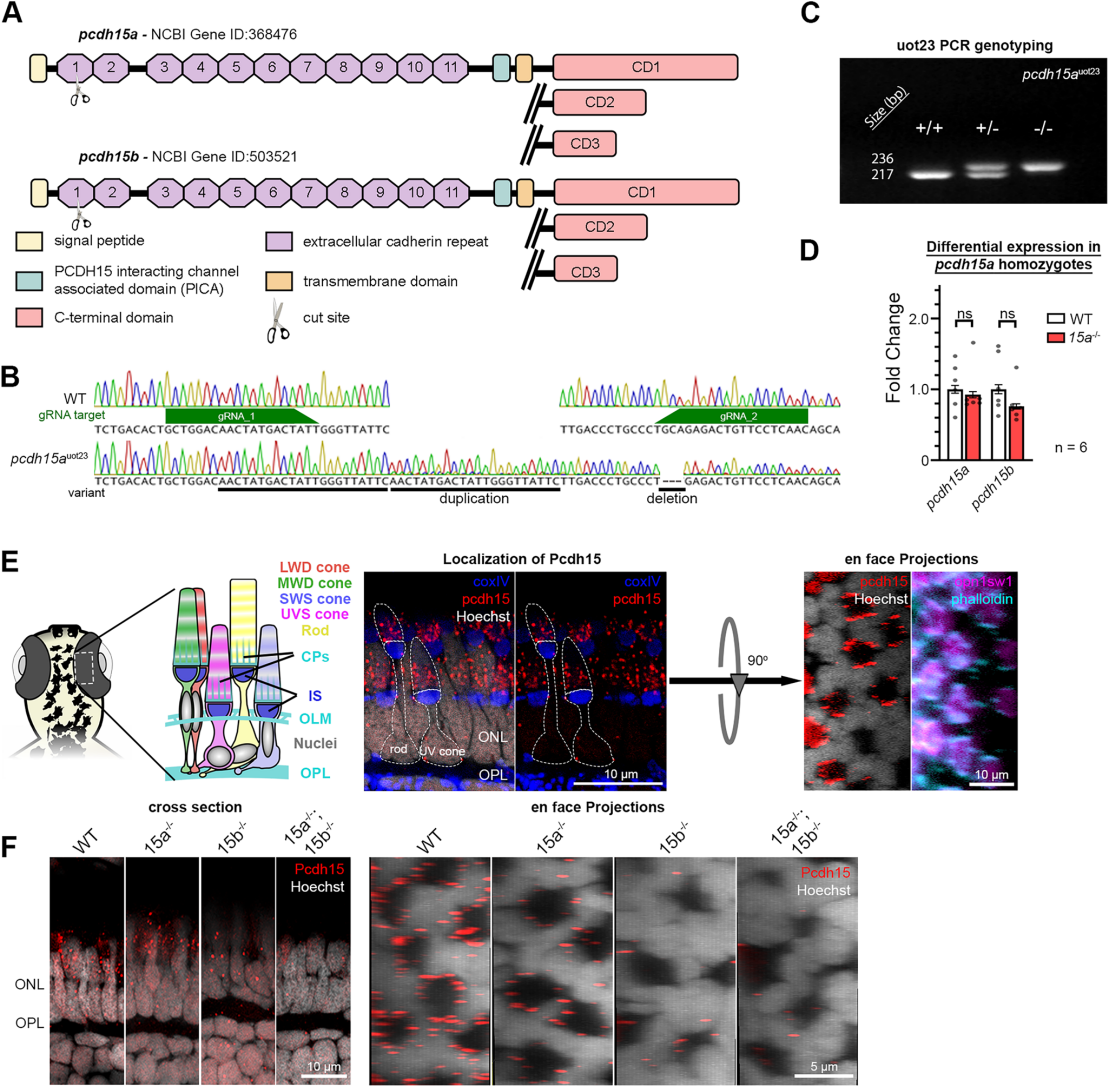
Generation and characterization of the *pcdh15a*^uot23^ mutant allele. **A** CRISPR gRNAs were targeted to the 5th coding exon of *pcdh15a,* which contributes to the first extracellular repeat domain of the protein. This methodology aligns with the approach taken previously to generate the *pcdh15b*^uot15^ allele. **B** Chromatogram from Sanger sequencing of the *pcdh15a*^uot23^ allele. Introduction of a 22 bp duplication and 3 bp deletion results in a frame-shift. **C**) The *pcdh15a* mutant band is readily resolved by agarose gel electrophoresis. **D** In 8 dpf mutant larvae there is no evidence of nonsense-mediated decay of the *pcdh15a* mRNA, or upregulation of *pcdh15b* (*P* = 0.6734, *P* = 0.1989, unpaired T-test). **E**) Schematic representation of the cellular architecture of the photoreceptor layer in 8 dpf retina. Protocadherin related 15 localizes to the proximal outer segment in both rows of photoreceptor cells. *En face* projections reveal Pcdh15 localizes around the whole circumference of the outer segment, consistent with the F-actin staining pattern. **F** The abundance of protocadherin related 15 puncta is reduced in *pcdh15a* and *pcdh15b* double mutants and are absent in double mutants. LWD = long wavelength double; MWD = medium wavelength double; SWS =  short wavelength single; UVS = UV wavelength single; CP = calyceal process; OLM = outer limiting membrane; OPL = outer plexiform layer; IS = inner segment; ONL = outer nuclear layer.

Having observed high levels of co-expression by *pcdh15* paralogs in the sense organs, we next sought to generate a double mutant with complete loss of protocadherin related 15 function more aligned to USH1F patients. Using a strategy similar to that utilized for the *pcdh15b*^uot15^ allele, we injected two gRNAs targeting the 5^th^ coding exon of *pcdh15a* in a region encoding the first extracellular cadherin repeat domain (Fig. 4A). This domain is critical for homodimeric binding to extracellular partners^41^. We recovered a complex mutation (22 bp duplication, 3 bp deletion) designated *pcdh15a*^uot23^ (Fig. 4B). This mutation (NM_001012482.1:c.253_274dup;289_291del; NP_001012500.1:p.(Leu92GlnfsTer3)) introduces a frameshift and premature termination codon within three residues and can be resolved by standard gel electrophoresis (Fig. 4C). There is no evidence of this mutation causing nonsense-mediated decay, since *pcdh15a* mRNA abundance was unchanged. Consistently, we observed no evidence of transcriptional adaptation by *pcdh15b* (Fig. 4D).

To ensure that the *pcdh15* mutants represented a complete loss-of-function, we next performed immunohistochemistry on tissues from the retina. In the central retina of 8 dpf larvae, there are terminally differentiated photoreceptors of all cone subtypes and rod cells. These photoreceptors form two rows with UV and short wavelength cones found basally and medium/long wavelength double cones and rods projecting more apically (Fig. 4E schematic). Protocadherin related 15 localized as small puncta in the photoreceptor outer segment layer, consistent with previous reports in human and macaque^33^. By creating *en face* (orthogonal) projections of z-stacks, we found that these puncta line the circumference of the photoreceptor outer segment and are consistent with F-actin staining around the outer segment circumference. This localization pattern is consistent with the coverage of the calyceal processes around the OS. In both *pcdh15a*^−/−^ and *pcdh15b*^−/−^ single mutants we observed a reduction in protocadherin related 15 expression in the retina (Fig. 4F). In *pcdh15a*^−/−^;*pcdh15b*^−/−^ double homozygotes, there was complete abolition of the protocadherin related 15 signal, revealing these alleles to likely be nulls.

### Both *pcdh15* paralogs have a role in zebrafish mechanosensation

**Fig. 5:**
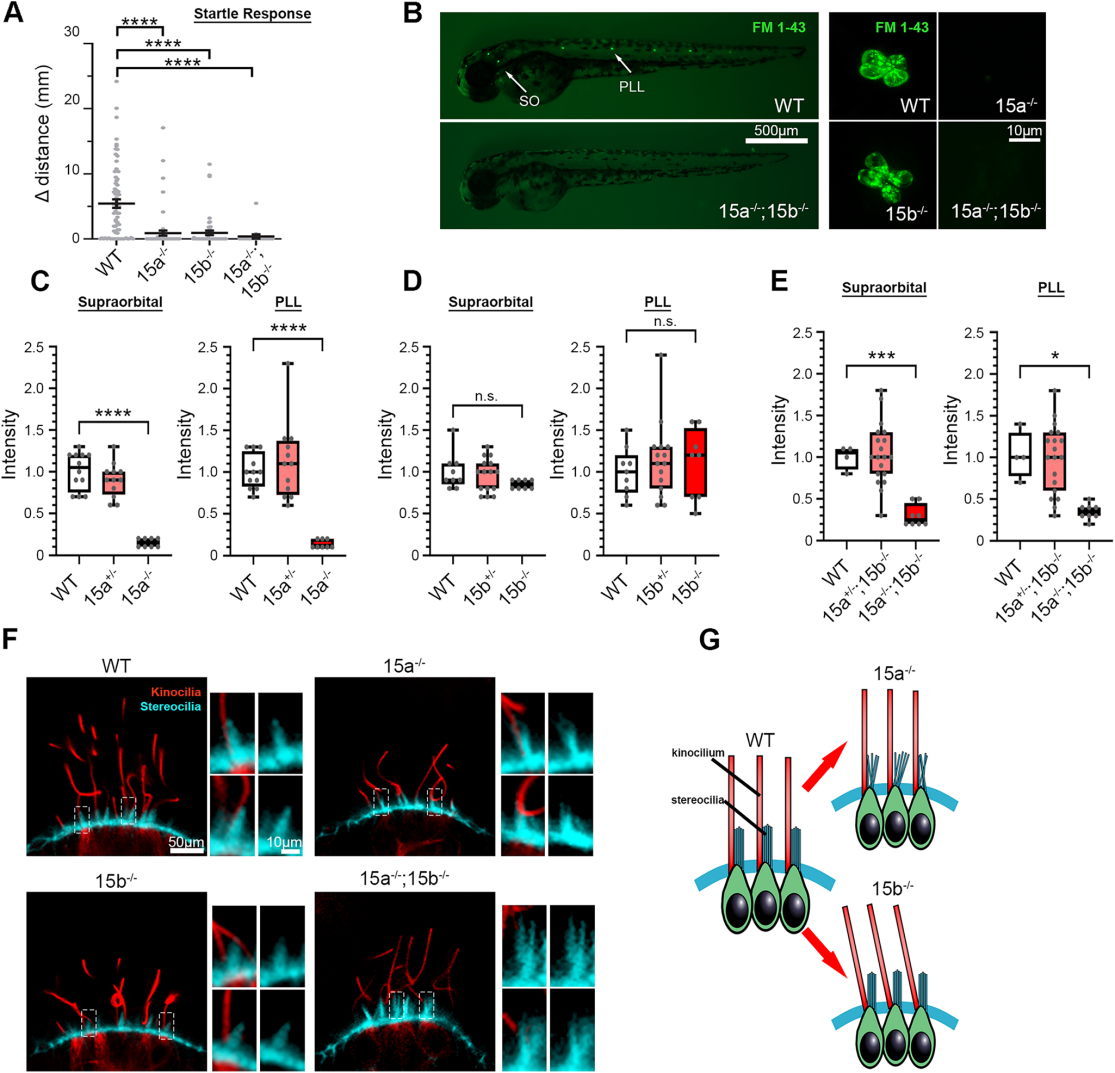
Both *pcdh15* paralogs have roles in the mechanosensitive organs of zebrafish. **A** Both single and double *pcdh15* homozygotes display dampened responses to an abrupt startle stimulus (*P* < 0.0001, all groups; Brown-Forsythe and Welch one-way ANOVA; *n*-value = 73, 60, 51, 17). **B**) Micrographs demonstrating the ability of neuromasts in the single and double mutants to uptake FM 1-43 dye through open MET channels. **C** *pcdh15a*^−/−^ homozygotes fail to uptake FM1-43 dye (SO *P* = 0.3997 het, <0.0001 homo; PLL *P* = 0.6773 het, <0.0001 homo; Brown-Forsythe and Welch one-way ANOVA; *n*-value = 12, 12, 8). **D** Inheritance of two mutant *pcdh15b* alleles does not impair uptake of FM1-43 (SO *P* = 0.8807 het, 0.1424 homo; PLL *P* = 0.5897 het, 0.8196 homo; Brown-Forsythe and Welch one-way ANOVA; *n*-value = 9, 15, 8). **E** Genetic dosage experiments demonstrate that only Pcdh15a is necessary for dye uptake; *pcdh15a*^+/−^; *pcdh15b*^−/−^ mutants shows no significant difference to WT controls, but *pcdh15a*^−/−^; *pcdh15b*^−/−^ double homozygotes had significantly reduced dye uptake (SO *P* = 0.9121 15a^+/−^; 15b^−/−^, 0.0003 15a^−/−^; 15b^−/−^; PLL *P* = 0.9732 15a^+/−^; 15b^−/−^, 0.0341 15a^−/−^; 15b^−/−^; Brown-Forsythe and Welch one-way ANOVA; *n*-value = 4, 19, 8). **F** Immunofluorescent micrographs of the 4 dpf medial crista stained for the kinocilia (acetylated tubulin) and stereocilia (actin). Inserts highlighting the splayed stereocilia of *pcdh15a*^−/−^ homozygotes and detached kinocilia of *pcdh15b*^−/−^ homozygotes. **G** Model representing the paralog-specific roles of Pcdh15 in the inner ear. *Pcdh15a* is necessary for attachment of adjacent stereocilia; *pcdh15b* is necessary for attachment of the kinocilium to stereocilia bundles. SO = supraorbital; PLL = posterior lateral line.

From the first generation ENU screens, it was discovered that *pcdh15a* mutants have reduced mechanosensation, leading to a failure to react to vibrational stimuli^38^. However, previous publications have not reported any changes to mechanosensation in *pcdh15b* mutants. We therefore tested mechanosensation of our *pcdh15* mutants using a startle response assay. An acousto-vibrational stimuli was propagated through the water via dropping of a 6 g weight to elicit a startle reflex in 6 dpf larvae. In both single and double *pcdh15* mutants tested we observed a significant reduction in the fast, darting swimming behaviour typical of the reflex (Fig. 5A). This result demonstrates that both *pcdh15a* and *pcdh15b* paralogs are required for eliciting a normal startle reflex.

Next, we wanted to perform functional testing of the lateral line mechanosensitive organs via FM1-43 dye uptake experiments. FM1-43 is a lipophilic styryl molecule that is impermeable to cells and non-toxic. However, upon bathing larval zebrafish, the dye will enter neuromast cells via open MET channels on the stereocilial tips (Fig. 1B)^42,43^. By measuring the difference in fluorescence (ΔF) of neuromasts compared to the surrounding tissue we have a readout for neuromast function. Consistent with previous reports, *pcdh15a*^−/−^ homozygotes fail to uptake fluorescent dye (Fig. 5B, C). However, *pcdh15b*^−/−^ homozygotes displayed no difference in dye uptake compared to WTs (Fig. 5D), consistent with a previous morpholino *pcdh15b* knockdown model^24^. Interestingly, *pcdh15a*^+/−^; *pcdh15b*^−/−^ larvae, which possess three mutant alleles, did take up dye at WT levels, whereas double homozygotes failed to uptake FM1-43 (Fig. 5E). Taken together, these data demonstrate that *pcdh15a* is necessary and sufficient for gating the neuromast MET channels, likely due to its contribution in forming the tip-link.

To determine whether all zebrafish mechanosensitive organs rely solely on *pcdh15a* function, we next examined the histology of the inner ear hair cells. The medial cristae of the inner ear cells project large kinocilia and bundles of stereocilia into the semicircular canal to detect body position^44^. Wholemount staining with acetylated tubulin and fluorescently conjugated phalloidin can reveal these features. Consistent with previous reports, we found that *pcdh15a*^−/−^ homozygotes had stereocilial bundle splaying (Fig. 5F top right), but we are the first to report that kinocilia remain attached to these splayed stereocilia. Interestingly, in *pcdh15b*^*−/−*^ homozygotes we see the opposite phenotype; stereocilia bundles remained intact but the kinocilium was detached with complete penetrance (Fig. 5F bottom left). Finally, *pcdh15a*^−/−^; *pcdh15b*^−/−^ double mutants displayed disconnection of the kinocilium and more severe splaying of the stereocilia. These data reveal a previously uncharacterized parsing of ancestral function in the *pcdh15* paralogs (Fig. 5G). Protocadherin related 15b is necessary and sufficient for the kinocilial links of inner ear hair cells. For the stereocilial links, the paralogs both contribute such that loss of both paralogs causes the most severe splaying phenotype. However, loss of *pcdh15b* alone is insufficient for splaying of stereocilia during larval stages.

### *pcdh15* mutants have visual function deficits

**Fig. 6:**
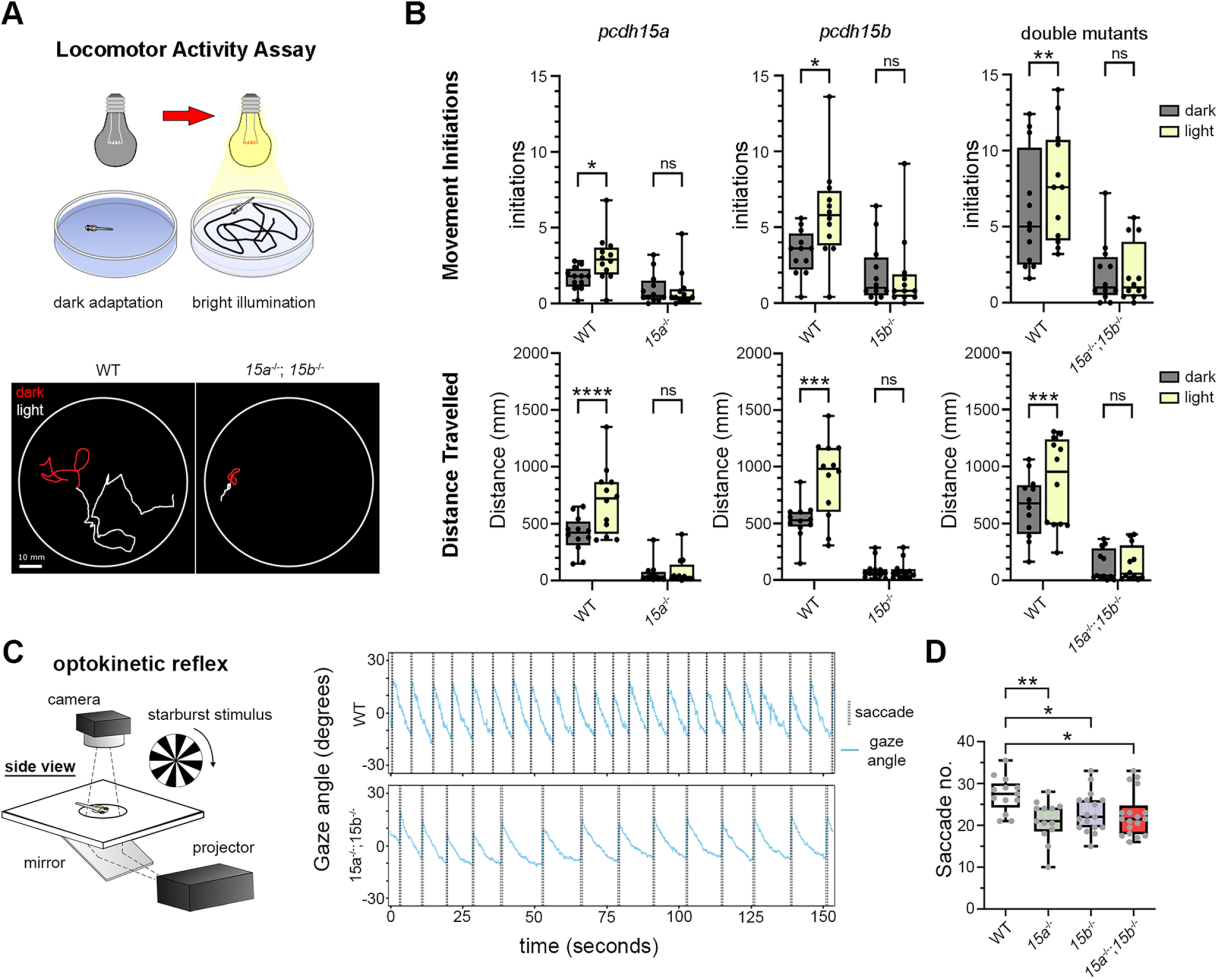
Reduced responses to visual stimuli in both single and double *pcdh15* mutants. **A** Schematic of the locomotor acuity assay and representative raw traces. 6 dpf larvae were dark-adapted and then exposed to a bright light stimulus. Locomotion was compared 2.5 min before and after the light switch. **B** In all tests, the WT animals increased the number of initiations and distance travelled following light switching. In contrast, there was no difference in behaviour in the *pcdh15a*^−/−^; *pcdh15b*^−/−^, or *pcdh15a*^−/−^;*pcdh15b*^−/−^ double homozygotes (*P* values in Table S3. two-way ANOVA Šídák’s post-hoc test; *n*-value = 12). However, swimming distance was lower in all mutant groups regardless of light intensity (Figure S3). **C** Schematic of OKR assay and representative traces. Larvae were immobilized in agarose with their eyes free to move. A black-and-white starburst stimulus was projected into the full field of vision and rotated at 10°/s. Eye movement was recorded from above. **D** The number of saccades performed over a 3-min test was compared between genotypes and were reduced in *pcdh15a*^−/−^; *pcdh15b*^−/−^, or *pcdh15a*^−/−^;*pcdh15b*^−/−^ double homozygotes (*P* = 0.0052 *15a*^−/−^, 0.0429 *15b*^−/−^, 0.0285 *15a*^−/−^;*15b*^−/−^. Brown-Forsythe and Welch one-way ANOVA, *n*-value = 13, 13, 17, 18).

Visual function was next assessed within the *pcdh15* mutants via locomotor activity assays. Larvae at 6 dpf were dark adapted for 20 min until locomotor activity stabilized, and then a bright light source was suddenly turned on (Fig. 6A). Under this light-switching paradigm, WT animals significantly increased locomotor activity in response to light stimuli. In contrast, the number of movement initiations, and the distance travelled did not change in *pcdh15a*^−/−^, *pcdh15b*^−/−^, or *pcdh15a*^−/−^;*pcdh15b*^−/−^ double homozygotes, consistent with a visual function deficit (Fig. 6B). One potential caveat was that both *pcdh15* single and double homozygotes also displayed reduced locomotor activity under stable lighting conditions (Figure S3). We therefore performed optokinetic reflex (OKR) assays to determine visual perception independent of swimming competency.

In this classical visual function test, the 6 dpf larvae were restrained to prevent body repositioning, and then a black-and-white starburst grating stimuli was rotated clockwise at 10°/s across the animal’s full field of vision. The eyes followed the stimuli rotation in a period of smooth tracking until the limit of abduction was reached, and a fast, counterclockwise saccade was registered (Fig. 6C). Although all genotypes were capable of tracking the stimulus, the number of saccades registered was reduced in single *pcdh15a*^−/−^ or *pcdh15b*^−/−^ homozygotes as well as *pcdh15a*^−/−^;*pcdh15b*^−/−^ double mutants (Fig. 6D). Taken together, these data suggest that both single and double *pcdh15* homozygotes have visual deficits compared to controls.

### *pcdh15* mutant photoreceptors have detachment of the IS/OS boundary, bending of the OS and disorganized actin processes

Our lab has previously demonstrated that *pcdh15b*^−/−^ homozygotes display an early dysmorphology within the cone outer segments, with abnormal disk growth, and partial detachment of the cone outer segment^26^. However, we did not detect any OS bending or overgrowth as reported in the *Xenopus* knockdown model^45^. To characterize the retinal phenotype of our double mutants we used transgenic zebrafish lines that specifically label UV cone and rod photoreceptor subtypes.

**Fig. 7:**
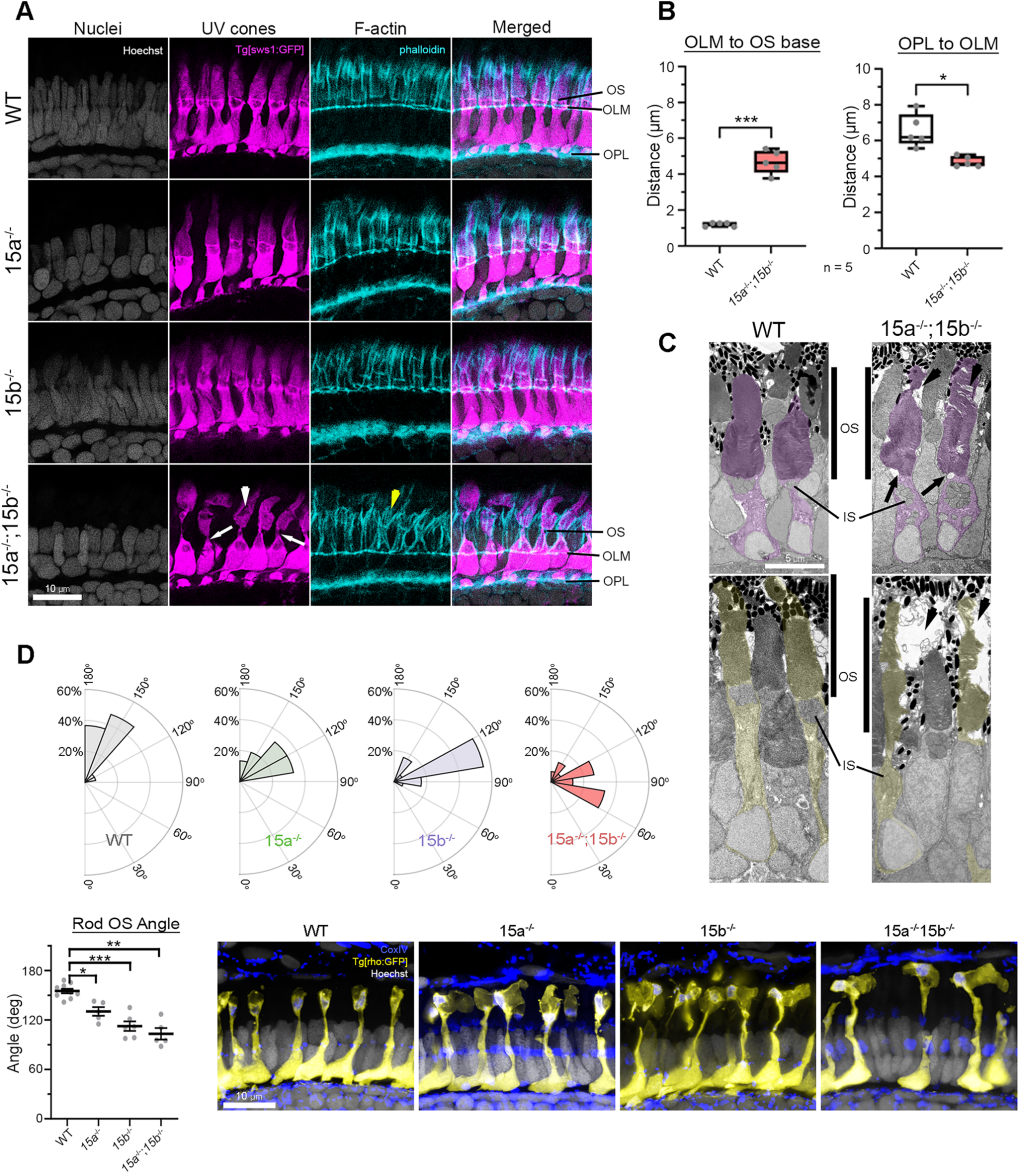
Loss of both *pcdh15* paralogs causes a more severe retinal phenotype. **A** Representative confocal micrographs of the *pcdh15* mutants on the UV cone-specific transgenic background. Double mutants present with holes/gaps in the outer segment staining (arrowhead) and separation of the inner segment/outer segment boundary, presenting as pinching of the cell (arrows). **B** The OLM is displaced basally, towards the OPL, while F-actin situated at the base of newly forming OSs increases in distance from the OLM (accompanying graphs). The actin filopodia surrounding the UV cones is ordered with parallel apical projections in WT animals but becomes disorganized and diverging in double mutants, following the contours of the cell (*P* = 0.0002; *P* = 0.0112 respectively; Welch’s T-test. **C**) Representative TEM micrographs corroborating the gaps in OS disks and separation of the IS/OS boundary (pseudocoloured to highlight UV cone and rod photoreceptors as magenta and yellow respectively). **D**) Analysis of rod photoreceptors revealed that the OS of single and double mutants was overgrown and bent at more acute angles than in WT controls. Radial histograms show the percentage of rod OSs in bins of 20°. Plotting the average angle per animal on a linear X-axis reveals that all groups (single and double mutants) are significantly different from WT controls (*P* = 0.0156 15a^−/−^, 0.0008 15b^−/−^, 0.0026 15a^−/−^;15b^−/−^ Brown-Forsythe and Welch ANOVA, *n*-value = 10, 5, 6, 5).

Despite no difference in photoreceptor abundance (Figure S4), the *pcdh15a*^−/−^; *pcdh15b*^−/−^ double homozygotes had a very severe UV cone dysmorphology at 8 dpf (Fig. 7A). There were apparent holes (white arrowhead) and horizontal bending of the outer segments within the plane of the tissue. Strikingly, there was also a “pinching” of the cell (white arrow) at the boundary between the inner and outer segments. This feature appears to be caused by an almost complete disconnection of the inner and outer segments of the cell, with the thin bridge that remains representing the connecting cilium. The IS of UV cones is identifiable in Tg[opn1sw1:EGFP] animals due a lack of cytoplasmic fluorescence where mitochondria are densely packed. The IS of double mutants was more basally located than in WT controls, closer to the synapse at the outer plexiform layer. Since the outer limiting membrane is located at the base of the IS, we used this landmark to measure the distance between the IS and OPL. In double mutants, the OLM to OPL distance was ~25% shorter than in WT controls (Fig. 7B left). Conversely, the distance between the OLM and the actin band at the proximal edge of the OS was almost 4 times longer in double mutants compared to WT controls (Fig. 7B right). These data validate the OS/IS boundary detachment resulting in basal displacement of the IS in *pcdh15a*^−/−^; *pcdh15b*^−/−^ double homozygotes.

Phalloidin staining was utilized to visualize the actin processes of the retina. Surprisingly, CPs were still present in *pcdh15a*^−/−^;*pcdh15b*^−/−^ double homozygotes and were observed surrounding the cone OS (Fig. 7A yellow arrowhead). However, the processes in double mutants were significantly disorganized. The straight and parallel arrangement of processes in the WT animals was replaced by bent and disorganized processes in the double mutants. TEM analysis enabled us to confirm the dysmorphology observed by immunofluorescent microscopy. Electron micrographs confirm that double mutant outer segments have gaps between disks and abnormal morphology (Fig. 7C arrowheads), with disconnection of the IS/OS boundary (Fig. 7C arrows) and basal migration of mitochondria in the IS away from the boundary with the OS.

Since the human phenotype of Usher syndrome is a rod-dominant degeneration we also examined the histology of rod photoreceptors using the Tg[rho:EGFP] line. The rod OSs at 8 dpf were overgrown in single and double homozygotes, with bending of the OS parallel to the plane of the tissue (Fig. 7D). Using the GFP expression of the Tg[rho:EGFP] line and CoxIV to mark the IS, we calculated the average angle of rod photoreceptors within the central retina. In single *pcdh15a*^−/−^, *pcdh15b*^−/−^, and *pcdh15a*^−/−^;*pcdh15b*^−/−^ double homozygotes there was significant deviation in the angle of the rod OS compared to WT controls, with a trend to greater severity in *pcdh15b*^−/−^ and double homozygotes. Whether this bending is due to loss of support from calyceal processes or is a result of the disk overgrowth remains to be elucidated. Interestingly, we found no evidence of IS/OS disconnection in rod cells, suggesting that the molecular underpinnings of this intersection may be different in zebrafish rod and cone photoreceptors.

### Severity of retinal phenotype is dependent upon *pcdh15* genetic dosage

**Fig. 8:**
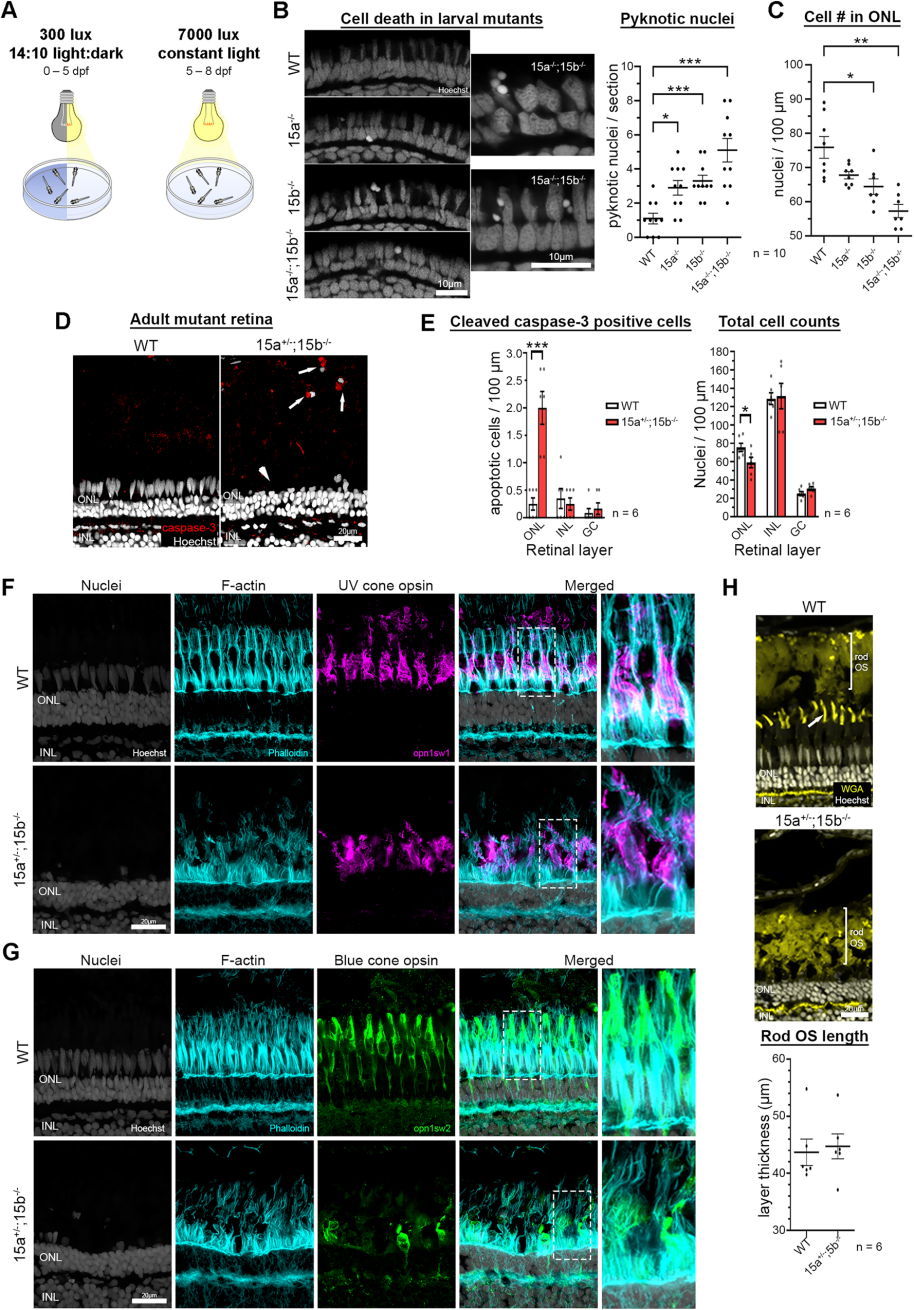
Cell death and dysmorphism of double mutant photoreceptors. **A** High-intensity light trial protocol. Larvae were reared under standard light:dark conditions until 5 dpf and then exposed to constant 7000 lux illumination for 72 h. **B** Following light trial, an increase in pyknotic cells of the ONL was observed in single and double homozygotes (*P* = 0.0116 15a^−/−^, 0.0004 15b^−/−^, 0.0004 15a^−/−^;15b^−/−^; Brown-Forsythe and Welch ANOVA). **C** Significant reduction in the number of ONL cells was measured in *pcdh15b*^−/−^ and *pcdh15a*^−/−^; *pcdh15b*^−/−^ double homozygotes (*P* = 0.1119 *15a*^−/−^, 0.0366 *15b*^−/−^, 0.0012 *15a*^−/−^;*15b*^−/−^; Brown-Forsythe and Welch ANOVA, *n* vaule = 8, 8, 7, 7). **D** Representative confocal micrograph of apoptotic photoreceptors in an 18 mpf *pcdh15a*^+/−^; *pcdh15b*^−/−^ mixed zygosity double mutant retina. **E**) Quantification of the increased number of apoptotic cells (*P* = 0.0012; Welch’s *T*-test) and decrease in absolute ONL nuclei number (*P* = 0.046; Welch’s *T*-test) in these mutants. **F** Representative micrographs of the UV cones and F-actin in the mixed zygosity double mutant retina. The UV cones are severely dysmorphic and fail to form the typical conical OS shape. Actin processes are sparse and disorganized. **G** Representative micrographs of the blue cones and F-actin reveal how sparse blue cones have become. The remaining blue cones are dysmorphic, with very little remaining OS projecting above the ONL. Gaps in the actin processes coincide with where cells have been lost. **H** Representative WGA staining revealed loss of cones (arrow) but normal thickness of the rod OS layer in mixed zygosity double mutants (*P* = 0.7495; Welch’s *T*-test).

Even with modified husbandry protocols, *pcdh15a*^−/−^; *pcdh15b*^−/−^ double homozygotes die around 14 dpf, likely due to complete penetrance of failed swim bladder inflation. The swimming behaviour of these larvae is also uncoordinated, and it is unlikely that they can feed effectively (Video S1). We therefore wondered if an increased cell stress paradigm via constant, bright light exposure could resolve differences in fitness between photoreceptors of the *pcdh15* mutant larvae. Animals were raised under standard light:dark conditions until 5 dpf and then transferred to constant 7000 lux illumination for 72 h prior to sacrifice at 8 dpf (Fig. 8A). Under these conditions, we observed a significant increase in ONL pyknotic nuclei in all mutant groups compared to WT controls, with double homozygotes having the greatest number (Fig. 8B). Performing cell counts of the ONL nuclei (Fig. 8C) we found a trend for the number of photoreceptors where WT > 15a^−/−^ > 15b^−/−^ > 15a^−/−^15b^−/−^ although the difference in 15a^−/−^ ONL nuclei did not reach statistical significance (*P* = 0.1119). In both analyses, mutant allele inheritance appears additive, with double homozygotes having a more severe phenotype than single mutants. These data represent the first reports of photoreceptor cell death attributable to a USH1F animal model, but require a paradigm that artificially stresses the retina.

Since double homozygote larvae do not reach adulthood, we next examined *pcdh15a*^+/−^; *pcdh15b*^−/−^ mutants, where a single WT *pcdh15a* allele is retained. These mixed zygosity double mutants are viable and fertile as adults without detectable differences and were raised under standard lighting conditions. However, as they age beyond ~15 mpf, these mutants begin to display uncoordinated swimming behaviour and struggle to maintain neutral buoyancy (Video S2). Since equilibrium orientation requires both optic and equilibrioception stimuli^38^, this may indicate progressive deterioration of vision or mechanosensation.

Focusing on retinal phenotypes, we collected *pcdh15a*^+/−^; *pcdh15b*^−/−^ mutants at 18 mpf and age-matched WT controls. Mixed zygosity double mutants displayed evidence of severe retinal dystrophy with loss of most blue, and double cone nuclei from the ONL (Fig. 8D arrowhead). We frequently observed nuclei detached from the ONL and displaced amongst the photoreceptor outer segments (Fig. 8D arrows). These nuclei were positive for the apoptotic marker cleaved caspase-3, suggesting that these were dying photoreceptor cells. Quantifying the number of apoptotic cells, we found an ~8-fold increase in the *pcdh15a*^+/−^; *pcdh15b*^−/−^ mutant ONL and a corresponding decrease in the absolute number of ONL cells (Fig. 8E).

Examination of photoreceptor morphology via immunofluorescence microscopy also revealed severe dysmorphology. The outer segments of both UV (Fig. 8F) and blue (Fig. 8G) cones were highly irregular and disordered compared to controls. The actin processes around these cells were highly disorganised with large gaps, completely lacking F-actin. The blue cones were more severely affected than UV cones in both number and length of the outer segment (Figure S5A, B), which is consistent with the almost complete lack of blue cone nuclei in the apical ONL. The severity of this retinal dystrophy contrasts with that observed in single *pcdh15b*^−/−^ homozygotes (Fig. 2), suggesting a strong genetic dosage effect; loss of one *pcdh15a* allele exacerbates the *pcdh15b*^−/−^ homozygote phenotype. Staining *pcdh15a*^+/−^; *pcdh15b*^−/−^ mutant sections with WGA, we confirmed the loss of cone outer segments in the central retina (Fig. 8H arrow), but surprisingly do not observe a change to thickness of the rod OS layer. This suggests that rod cells are either less susceptible to cell death in zebrafish USH1F models (contrary to human disease) or that rods are being replenished by the nascent rod progenitor cell population^46,47^. Taken together, these data reveal that double mutants have more severe retinal phenotypes than single *pcdh15b*^−/−^ homozygotes, with susceptibility to cell death following either cell stress, or during normal aging.

### Isoform-specific roles for *pcdh15*

**Fig. 9:**
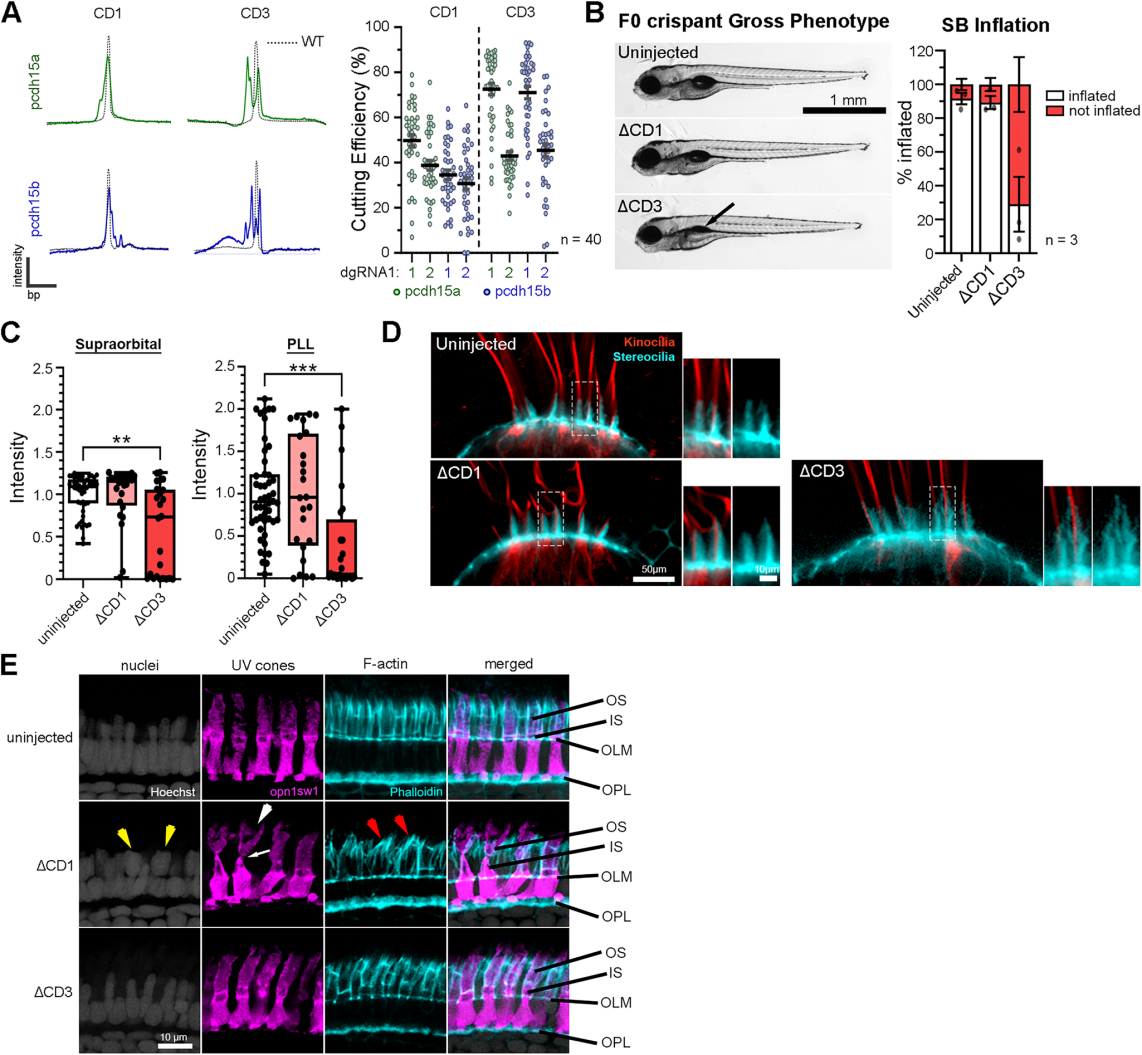
Evidence of an isoform-specific role for the *pcdh15* paralogs. **A** Representative traces of WT amplicon mobility (dotted line) Vs the population of amplicons generated in *pcdh15a* (green) or *pcdh15b* (blue) CD1 and CD3 dgRNA injected embryos at 5 dpf. Column graph shows the mean cutting efficiency and variability of indels introduced with each dgRNA. **B** F0 CRISPR injected larvae were indistinguishable from uninjected controls, except for a lack of swim bladder inflation in the ΔCD3 animals (*n* = 3 clutches. Uninjected 146, ΔCD1 161, ΔCD3 104 animals respectively). **C** FM1-43 dye uptake was normal in ΔCD1 larvae but significantly reduced in ΔCD3 CRISPR injected (SO *P* = 0.9999 ΔCD1, 0.0015 ΔCD3; PLL *P* > 0.9999 ΔCD1, 0.0005 ΔCD3; Brown-Forsythe and Welch one-way ANOVA; *n*-value = 48, 24, 24). **D** Representative confocal micrographs revealed that the medial cristae of ΔCD1 larvae were indistinguishable from controls, but in ΔCD3 CRISPRants, the stereocilia were splayed while the kinocilium remained attached. **E** *pcdh15* ΔCD1, but not ΔCD3 CRISPants display photoreceptor dysmorphology. The UV cones (Tg(-5.5opn1sw1:EGFP)kj9) of ΔCD1 injected embryos have disconnection of the IN/OS boundary (arrow), holes in the OS (white arrowhead), disorganized actin processes (red arrowhead) and abnormally shaped nuclei (yellow arrowhead). OS = outer segment ; IS = inner segment; OLM = outer limiting membrane; OPL = outer plexiform layer.

We previously demonstrated that the major contribution of *pcdh15* expression in the eye comes from -CD1 expressing isoforms of *pcdh15a* and *pcdh15b*, whereas the -CD3 isoforms are most abundant in the ear (Fig. 3C+E). To investigate if this expression pattern divergence was indicative of isoform-specific functionality we attempted to knock out the isoforms individually. Two duplex guide RNAs (dgRNAs) were designed against the -CD1 encoding exon and the -CD3 encoding exon of each paralog. Two, non-overlapping ( > 300 bp separation) dgRNAs were used to maximize the probability of mutagenesis ubiquitously throughout the body^48^. Cutting efficiencies were determined for each dgRNA via quantification of the WT locus relative to indels created (Fig. 9A and Figure S6A). Using this method, we routinely found cutting efficiencies between 30 – 70% per dgRNA, at doses that caused <20% developmental dysmorphology (Figure S6B). Since two non-overlapping dgRNAs were administered per isoform, this combinatorial approach was suitable for generating loss-of-function F0 animals (crispants).

The ΔCD3 larvae were identifiable due to a failure to inflate the swim bladder by 5 dpf, whereas ΔCD1 larvae were indistinguishable from uninjected controls (Fig. 9B). The ΔCD3 larvae also displayed deficiencies in neuromast mechanoelectrical transduction as assessed via FM1-43 dye uptake (Fig. 9C). Examination of the medial cristae revealed that ΔCD3 larvae had splayed stereocilia but attached kinocilia. The ΔCD1 larvae had no detectable differences in hair cell morphology (Fig. 9D). These data are consistent with the idea that the -CD3 containing isoforms are utilized in mechanosensation, whereas -CD1 isoforms are dispensable. Conversely, retinal histology was abnormal in ΔCD1 larvae but indistinguishable in ΔCD3 animals. Assessment of the ONL revealed that nuclei of the photoreceptors were rounded and more closely packed in ΔCD1 crispants (Fig. 9E yellow arrowhead). The UV cones appeared pinched and almost completely detached at the IS / OS boundary with gaps in the staining of the OS (white arrow, white arrowhead respectively). Finally, the F-actin apical processes were disorganized and had lost their parallel arrangement in ΔCD1 retinas (Fig. 9E red arrowheads). These data confirm that there are isoform-specific roles of protocadherin related 15 in the sense organs; the -CD1 isoforms are required for development of the photoreceptor cell morphology, whereas the -CD3 isoforms are required for development and function of the mechanosensitive hair cells of the lateral line and ear.

## Discussion

In this study we have generated the first zebrafish double mutants for any Usher syndrome gene and demonstrated that both *pcdh15a* and *pcdh15b* paralogs contribute to the development and function of mechanosensation and vision. In contrast to previous studies, we show that there is not a dichotomic divergence in zebrafish paralog expression, denoting *pcdh15a* as “ear specific” and *pcdh15b* as “eye specific”. Instead, loss of both paralogs is required to detect the most severe phenotypes in either tissue. In the hair cell of the ear, loss of both paralogs results in the most severe splaying of the stereocilia plus detachment of the kinocilium. In retinal photoreceptors, the loss of both paralogs is required to observe the most disorganized actin processes and detachment of the inner and outer segments. As adults, mixed zygosity *pcdh15a*^+/−^; *pcdh15b*^−/−^ double mutants show clear evidence of retinal degeneration, which is absent from *pcdh15b*^−/−^ single homozygotes. These data expand our understanding of the role of *pcdh15* in the ear and retina and highlight a possible confound for zebrafish models: where paralogous genes are retained there is potential for compensatory function when studying single mutants. We recommend generating double mutants (where possible) when studying genes that possess duplicates to determine if redundancies exist, and to more completely understand their function and divergent evolution.

Since the discovery of two duplicate *pcdh15* genes in the zebrafish, it was proposed that there was a subfunctionalization of the ancestral function^24^. From in situ hybridization (ISH) analysis, it was shown that the expression domain of *pcdh15b* was restricted to the neural retina and pineal organ (putatively expressed in the pineal photoreceptors^49^) with limited additional expression domains, whereas *pcdh15a* was expressed in the otic vesicle and neuromasts. However, upon re-examination of the sequence used to generate that *pcdh15b* ISH probe (AY772391.1) with contemporary refeq datasets, we find that the probe was complementary to the *pcdh15b*-CD1 isoform specifically (99.6% sequence identity Figure S7) and is therefore unlikely to represent pan-*pcdh15b* expression. In this context, the ISH data align with our own observations that *pcdh15b*-CD1 is: a) abundantly expressed in larval ocular tissue and b) not abundant in the otic vesicle. A subsequent ISH study also revealed that the *pcdh15a*-CD3 isoform has >2 fold greater expression in the adult ear than the -CD1 isoform^25^ (published prior to the annotation of *pcdh15a*-CD2). Our qRT-PCR analyses again align with these findings because -CD3 expression was persistently the most abundant isoform in ear tissues (Fig. 3E). However, we extend these findings by performing quantitative analysis of each isoform across development from embryonic to adult stages in the sense organs. From these data we have identified a contribution from both paralogs to both the ear and eye during WT development, determining that these paralogs function cooperatively and that double mutants are necessary to generate a complete *pcdh15* loss-of-function model. We have also shown that some isoforms are more abundant in the ear versus the eye, which may be related to differential function.

Protocadherin related 15 is an atypical cadherin thought to function as a cell adhesion molecule. In mechanosensitive hair cells of the inner ear, the extracellular cadherin repeat domains of protocadherin related 15 and Cadherin-related 23 form a handshake interaction that performs either structural or sensory functions^50^. At the tip link, this molecular bridge acts as a “mechanosensitive spring” to gate MET channel opening in response to shear forces^41^. Whereas in the transient lateral links and kinocilial links, this complex binds adjacent cellular projections together to form the staircase stereocilia arrangement (lateral) or pattern the planar polarity of the tissue (kinocilial) during development^20,22,51^. How these disparate functions are realized by a common gene is likely due to the differential splicing of the C-terminal cytoplasmic domain and by alternate cytoplasmic partner binding. The latter function likely reflects why the intracellular portion of each isoform has only 20 to 30% sequence identity (Fig. 1A).

Although all isoforms of *Pcdh15* have been detected in the cochlea^20^, evidence for isoform-specific roles is complex and sometimes contradictory. In isoform-specific murine mutant lines, the ΔCD1 and ΔCD3 knockouts do not cause hearing loss, whereas loss of ΔCD2 causes deafness^22^. Consistently, when the -CD2 domain was conditionally knocked out in post-natal hair cells, tip link formation was abolished^52^. These studies suggest that protocadherin related 15-CD2 may be the predominant tip link component, with other isoforms contributing to other links. However, alternative studies have found that, while ubiquitous ΔCD2 does cause polarity defects and hearing loss, tip link formation remains intact^22^. Yet further studies have shown that localization of the three isoforms places the -CD1 and -CD3 isoforms as the most likely components of the tip link^20^. The most parsimonious explanation for these discrepancies is that there is considerable functional redundancy that is built into the gene network, and that multiple isoforms are required for different development stages. Since *PCDH15* proteins dimerize through interaction of the extracellular cadherin repeats^53^, there could also be heterodimerization by alternate C-terminal isoforms to form these links. In our own analyses we find that isoform abundance can vary greatly during development, but all three isoforms were expressed to some extent in both the zebrafish ear and eye at different ages (Fig. 3).

The splaying of inner ear stereocilia is a common feature of zebrafish Usher syndrome mutants^24,54–56^. In this study we have shown that stereocilial splaying is attributable to loss of the -CD3 isoform in *pcdh15a/b* mutants (Fig. 8D). However, splayed cochlear stereocilia are not observed in any of the respective isoform-specific murine models^22,52^. Instead, mutations affecting all murine isoforms do cause stereocilial splaying and lead to eventual degeneration of the neuroepithelium^57–59^. These data demonstrate that there are species differences in phenotypes and caution direct extrapolation to human disease. Due to a paucity of human histopathological analyses before adulthood, it is unclear whether stereocilia splaying is also observed in the Usher syndrome patient cochlea. Where temporal bone dissections have been published, the only consistent finding was a neuroepithelial degeneration^60–65^, reminiscent of the aged murine cochlea. However, none of these patient dissections were performed before the second decade of life and may therefore represent late-stage disease of the tissue. Indeed, USH1F patients have congenital deafness suggesting that the cellular insult is developmental.

In contrast to vestibular hair cells, murine cochlear hair cells possess transient kinocilia critical during development^66–68^. These organelles help pattern the organ of Corti, and perturbation of the kinocilia disrupt planar polarity of the tissue^69,70^. In zebrafish, hair cells kinocilia persist into adulthood, but the interactions between kinocilia and stereocilia remain poorly characterized. To our knowledge, we are the first to report that loss of *pcdh15* causes detachment of the zebrafish kinocilium from the stereocilia, an effect attributable to the *pcdh15b* gene (Fig. 5F). Interestingly, neither the ΔCD1 nor ΔCD3 crispant models recapitulated this defect (Fig. 9D), suggesting that *pcdh15*-CD2 may be required for kinocilial links. Supporting this, murine ΔCD2 mutants display cochlea planar polarity defects^22,71^. However, we have not directly tested this hypothesis in zebrafish and can not discount contribution from multiple isoforms. It also remains to be elucidated whether kinocilial to stereocilial connection require *pcdh15b* in the neuromasts, and whether loss of *pcdh15b* affects hair cell planar polarity.

The most challenging aspect of studying Usher syndrome pathomechanism has been to understand the progressive vision loss of patients. Murine models of USH1 do possess mild visual deficits, but do not have pronounced histological changes or retinal dystrophy^35,36,72–77^. This contrasts with zebrafish, *Xenopus* and porcine models of USH1, where studies have shown significant retinopathy due to loss of USH1 genes^26,45,56,78,79^. It is proposed that this discrepancy is due to loss of the primary site of insult in nocturnal rodents. The Usher proteins localize to calyceal processes, which are filamentous actin-based processes that span the inner/outer segment boundary^31,32,34^. Although CPs are a feature of photoreceptors of most species, they have been lost in the nocturnal rodents^33^. These CPs are believed to play a structural role in the retina to support the long, elaborated cilia which form the outer segments. Our data demonstrate that protocadherin related 15 does indeed localize to the circumference of the basal outer segment (Fig. 4F), and we validate the antibody by showing abolition of the signal in double homozygotes. The localization pattern is coincident with actin apical processes around the photoreceptors, although direct co-localization cannot be assessed due to loss of phalloidin binding when antigen retrieval is performed. In double mutants, F-actin remains opposed with the OS; however, the inner and outer segments become disconnected, causing a pinching at the connecting cilium. From our isoform-specific crispants, we can attribute this phenotype to loss of the *pcdh15-CD1* isoform. Crispants also had a basal retreat (towards the OPL) of the inner segment, which forms the ellipsoid zone on clinical OCT scans^80^. Since contraction and discontinuation of the ellipsoid zone is an early indicator of retinal dystrophy in Usher syndrome patients^81–83^, further investigation of this phenotype may uncover disease pathomechanism.

In conclusion, our work challenges the notion of strict partitioning of function between the paralogous *pcdh15* genes via expression pattern divergence. While the *pcdh15* genes show some degree of subfunctionalization, it is necessary to knock out both genes to uncover the phenotype of the true null in either ear or eye tissues. Regardless of whether acting through genetic redundancy or transcriptional adaptation, there is a risk of masking gene function unless both copies are removed. In the era of high-efficiency genome editing by the CRISPR/Cas system, we hope that this study will encourage researchers to consider generating double mutants for the study of human monogenic diseases. Comparison of single to double mutants can provide insight into paralogs cooperativity and may help to uncover different facets of disease pathomechanism (e.g. kinocilial link versus stereocilial link disconnection). Through careful characterization, these studies can reframe a possible confound of the zebrafish genome (i.e. teleost WGD) into a positive trait for understanding gene function.

## Methods

### Fish husbandry

All experiments were performed in accordance with the regulations for animal experimentation established by the Canadian Council on Animal Care and approved by the University of Toronto Animal Care Committee, protocol #20011288. We have complied with all relevant ethical regulations for animal use. All fish used in this study were on the AB laboratory strain, studied at 2, 5, 8, 14 days or 1, 4, 8 or 18 months post fertilization. Sex of animals could not be determined prior to 4 mpf, and equal numbers of male and female animals were selected after 4 mpf. Transgenic and mutant lines used in this study are detailed in Supplementary Table 3.

### Retinal immunohistochemistry

Adult eyes were enucleated, lens dissected, and tissues fixed in 1 mL of 4% buffered paraformaldehyde (pH 7.4, dissolved in phosphate-buffered saline) overnight at 4 °C. Prior to cryopreservation, the adult eyes were bisected along the anterior-posterior axis. For larval fish, the tail was removed for genotyping, and the head was fixed as above. Tissues were then processed through a gradient up to 20% sucrose for cryopreservation. Tissues were embedded in a 2:1 mixture of 20% sucrose: Tissue-Tek® O.C.T. compound (Sakura Finetek 4583) and cryosectioned at 20 μm thickness. Slides were permeabilized in PBS-T (phosphate buffer saline + 1% Tween-20), blocked in 5 mg/ml bovine serum albumin, 5% normal goat serum, and probed overnight with 1:500 primary antibody in blocking agent at 4 °C. Following three PBS-T washes, slides were probed with 1:500 secondary antibody and/or fluorescent dye-conjugated phalloidin in blocking agent for 2 h. Slides were counterstained with 1:1000 Hoechst 33342 for 30 min, and mounted with ProLong™ Gold (ThermoFisher P36930). Z-stacks were imaged on the Leica SP8 confocal microscope, and maximum intensity projections were produced. For anti-Pcdh15 detection, an antigen retrieval step was added prior to blocking. Slides were incubated in 95 °C 10 mM sodium citrate, 0.05% Tween-20, pH 6.0 for 10 min. To control for globe eccentricity, all sections analysed were +/− one section from the optic nerve. Photoreceptors analyzed were within the central 1/3rd of the retina. Antibodies used can be found in Supplementary Table 3.

Quantification of nuclei in the neural retina was semi-automated using IMARIS (Oxford Instruments). Surfaces were manually drawn around the ONL, INL and RGC layer and masks of the Hoechst channel were created. The Spots tool was then used to automatically identify nuclei. Manual curation for some ONL nuclei was required in WT sections due to the elongated shape of cone nuclei, leading to double counting. The measurements of length and angle in photoreceptors were performed using the Measurements tool. Angle was determined from the most distal OS tip to the surface of the CoxIV signal, to the base of the cell body.

### Wholemount medial cristae immunofluorescence

For stereocilia and kinocilia imaging of the medial cristae we followed the protocol in Maeda, 2017^25^. Larvae were collected and fixed as above. The heads were permeabilized in PBS-Tx (PBS + 0.5% Triton X-100) for 1 h with 50 rpm shaking, and then overnight at 4 °C. The tissue was blocked for 2 hours as above, and then incubated in 1:200 anti-acetylated tubulin overnight at 4 °C. Following 3 ×1 h PBS-Tx washes, tissues were probed with 1:500 secondary antibody and 1:50 phalloidin. Heads were washed 3 ×1 h in PBS-Tx and then mounted in 1% LMP agarose (Fisher Scientific BP165-25). Z-stacks of the mechanosensitive hair cells of the medial cristae were imaged by confocal microscopy, and maximum intensity projections produced. Antibodies used can be found in Supplementary Table 3.

### Generation of *pcdh15a*^uot23^ line

CRISPR/Cas9 mutagenesis of *pcdh15a* was performed following a modified protocol found in the supplemental materials of Gagnon et al. 2014^84^. Two spacer RNA sequences were selected from the CHOPCHOP server^85^ to target the 5th coding exon of *pcdh15a*. The spacer sequences were flanked by an SP6 promoter and complementary sequence for the constant oligo. Spacer and constant oligos were ordered from IDT (sequences in Supplementary Table 3) and hybridized together and elongated using T4 DNA polymerase. sgRNAs were transcribed using the SP6 MEGAscript kit and mixed at 1:1 ratios for a final concentration of 300 ng/μl. 1 μL of EnGen Spy Cas9 NLS protein was added prior to injection into the one-cell stage zebrafish embryo. Successive outcrosses were performed to produce an F0 generation prior to usage. Sanger sequencing was performed to confirm the UoT23 allele sequence variation.

### gDNA extraction and PCR genotyping

PCR-ready genomic DNA was obtained from tissue by boiling for 20 minutes in 50 mM NaOH, vortexing vigorously, and neutralizing with 1 M Tris pH 8.0^86^. Larval tail biopsies were performed on anaesthetised in 55 µg/mL eugenol, and a scalpel was used to remove the tip of the tail, distal to the caudal blood vessels^37^. The larvae were moved to a 24-well plate of fresh E3 to recover, and the tail tissue was transferred to 20 µL NaOH and processed as above. Genotyping PCRs were performed using GoTaq G2 DNA Polymerase, and primer sequences can be found in Supplementary Table S3. Electrophoresis was performed in a 1–3% agarose gel at 250 V constant voltage using sodium boric acid electrophoresis buffer^87^.

### Transmission electron microscopy

Embryos were fixed in 2.5% glutaraldehyde in 0.1 M Sorensen’s Phosphate buffer pH 7.2 for >24 h at 4 °C. Tissues were washed 3 ×10 min in Sorensen’s buffer, and a secondary fixative step of 1% osmium tetroxide was performed for 1 hour. Following 3 × 10 min washes, the samples were serially dehydrated into 90% ethanol. Samples were transferred into Spurr epoxy resin and oriented for coronal sectioning before curing for 18 h at 65 °C. Ultrathin 100 nm sections were transferred to high transmission grids and stained with 3% uranyl acetate in 50% methanol for 45 min and Reynold’s lead citrate for 15 min in Sorensen’s Phosphate buffer es. TEM was performed on the Hitachi HT-7700 at 80 kV.

### RT-PCR and qRT-PCR

Eye and ear tissues were dissected using sharpened tungsten needles at the required stage from WT or mutant lines. Adult ears were dissected following removal of the brain using spring scissors and sharpened tungsten needles^88^. Where *pcdh15a* or *pcdh15b* homozygote tissue was used, the animals were genotyped by larval tail biopsy prior to dissection as above. Tissues were plunged immediately into 1 mL of TRIzol™ Reagent to create pooled samples: eyes at 3 dpf *n* = 50, 8 dpf *n* = 35, 14 dpf *n* = 35, 1 mpf *n* = 3, 4 mpf *n* = 2; ears at 4 dpf *n* = 50, 8 dpf *n* = 35, 14 dpf *n* = 15, 1 mpf *n* = 3, 4 mpf *n* = 2. The aqueous phase was removed following addition of 200 µL chloroform and centrifugation at maximum speed. Total RNA was extracted by precipitation in propanol, and rinsed with 1 mL 75% ethanol. 500 ng of total RNA was used for cDNA synthesis using SuperScript™ IV Reverse Transcriptase and diluted 1:5 prior to use. For endpoint reverse transcription PCR we used GoTaq® G2 DNA Polymerase. PCR was performed at 58 °C annealing temperature for 30 s elongation and with 35 cycles. Primer sequences are provided in Supplementary Table 3. Gel electrophoresis was performed using 1% agarose in SB buffer for 60 minutes at 150 V.

All quantitative real-time PCR (qRT-PCR) was performed following the recommendations of Taylor et al. 2019^89^ and validated using standard serial dilutions under MIQE guidelines^90^. All 10 µL qRT-PCR reactions were run in technical triplicate with ≥ 6 biological replicates using LightCycler® 480 SYBR Green I Master and normalized against the geometric mean of three housekeeping genes: β-actin, lsm12b, and mob4^91^. The Cq values were then normalized against the WT sample in Fig. 3A, *pcdh15a* 3dpf in Fig. 3B, *pcdh15a*-CD3 in Fig. 3C, *pcdh15b* 4 dpf in Fig. 3D, *pcdh15b*-CD2 in Fig. 3E, and the WT sample in Fig. 4D. All primer sequences are provided in Supplementary Table 3.

### Startle reflex testing

The Zebralab automated tracking system was used to track larval swimming behaviours in response to a startle stimulus. For each replicate, 6dpf WT (*n* = 6) and putative mutants (*n* = 18) were placed in 24-well plates and moved into the Zebrabox apparatus for an acclimation period of ≥20 min. Swimming behaviour was recorded for a 2-min period, with three stimuli provided at 30 s intervals. The startle stimulus was provided by dropping of a 6 g metal weight from a height of 15 cm onto the base holding the 24-well plate. The fast-swimming distance (in mm) was determined by the software and extracted as an Excel file. The trigger window was determined as a 5 s period, beginning at the trigger initiation time. The startle reflex was computed as the fast swim distance for 5 seconds post stimulus minus the fast swim distance for 5 s pre-stimulus. The distances were averaged across three replicates of startle stimulus. Any startle reflex values that were negative (i.e. larvae were engaged in fast swimming immediately prior to stimulus) were excluded from the analysis. Excluded sample number per group: 5, 1, 4, 2, respectively.

### Visual function testing

Locomoter activity under constant light or dark-to-light switching conditions was performed over 300 second recordings. Larvae at 6 dpf were transferred to a six-well plate below a suspended 5MP CMOS monochrome camera (Mightex), and illuminated from above by infrared light. Larvae were then acclimatized to the lighting conditions for 20 min prior to video capture. Videos were taken at 20 frames per second, with a 250 lux white light turned on after 150 s in the switching paradigm, or in constant illumination for the whole test. Following the recordings, animals were genotyped as above. Total distance travelled and movement initiations were calculated with FIMTrack^92^ and normalized over a 100 s duration.

For optokinetic reflex testing, a custom-developed web application *Socialize*, was used for this assay. Larvae at 6 dpf were embedded in 2.5% low melting point agarose, with agarose around the head dissected away to ensure free motion of the eyes. The agarose was submerged in E3 media and survival of the larvae was monitored throughout the duration of the test. Larvae were lowered into a 20 cm watchglass imaging chamber, and the visual stimulus was projected from below using the AAXA P8 Smart Mini Projector. Recordings of eye motion were taken from above using a BFS-U3-23S3M-C USB 3.0 Blackfly S monochrome camera, equipped with a Ricoh FL-CC1614A 2 m lens and a BP800-27 IR filter. A 15 cm diameter black and white sunburst disk stimulus with 20° wide segments was rotated clockwise at 10°/s. The response to the OKR test was recorded for 180 s at 30 fps. A pre-trained *DeepLabCut*^93^ network tracked the position of each eye frame by frame, labelling the anterior end, posterior end, and center of each eyeball. Eye-tracking data were processed using a custom analysis script to obtain eye-position traces. For each frame, head orientation was estimated from the midpoint of the two eyes, and a perpendicular reference vector was computed to normalize eye angle by head direction. For each eye, a linear fit to the labelled points defined the directional vector, and eye angle was calculated relative to the head reference using the vector dot-product formula.

Eye-angle traces were temporally smoothed using a Gaussian filter (SD = 4 frames). Eye velocity was quantified as the frame-to-frame change in eye angle, and saccades were identified using a median-absolute-deviation (MAD) outlier detection threshold (*K* = 5–10). High-velocity frames that exceed *K* * MAD were classified as saccades. Larvae that had no response, or did not complete the full 180 s OKR response trial were excluded from analysis.

### FM1-43 uptake assay

Zebrafish embryos were raised to 2 dpf in E3 media and dechorionated. FM1-43FX dye (ThermoFisher Scientific) was dissolved in 200 µL DMSO and then diluted to 15 µM in E3 media. Embryos were bathed in FM1-43FX working solution for 1 minute and then immediately washed 3 ×1 min in E3^94^. Embryos were then anaesthetised as above and imaged on a Lumar V12 (Zeiss) stereomicroscope with Axiocam 712 mono (Zeiss) camera. In FIJI^95^ the point tool was used to calculate the ΔFluorescence between neuromast cells and background.

### Bright light rearing

Zebrafish larvae were reared under standard conditions until 5 dpf. Between 5 and 8 dpf, the zebrafish were instead reared in a dish under constant 7000 lux illumination and covered by aluminium foil to reflect illumination. All other variables were unchanged. Daily health checks, feeding and water changes were performed as normal. After 72 h of illumination, larvae were sacrificed with head tissues being fixed and tails being used for genotyping as above. Sectioning and immunofluorescence were then performed as normal. Pyknotic nuclei were identified post-Hoechst 33342 staining as above. Pyknotic nuclei were identified by their condensed appearance: <50% area of healthy nuclei and greater signal intensity.

### Survival curve

For the survival curves, a *pcdh15b*^+/−^ incross was performed, and larvae were raised to 3 dpf. Larval tail biopsies were performed as described above and larvae were raised separately in a 96-well plate until genotyping was completed. The three genotypes (*15b*^+/+^, *15b*^+/−^, *15b*^−/−^) were then raised separately under standard conditions in the aquatics facility. Images of the nursery tanks were taken weekly to allow for counting of the fish and plot the survival curve.

### Statistics and reproducibility

All data were blinded prior to analysis and statistical tests were performed in GraphPad Prism V10. All experiments were performed at least twice on separate days. In all analyses the biological replicates are defined as different animals or different pools of tissue for qRT-PCR analysis. The number of biological replicates is provided in each figure panel or legend except where no quantification was performed. Where data was qualitative in nature we show a representative image from *n* = 3 replicates (validating the *pcdh15* antibody and electron microscopy) or *n* = 6 replicates (inner ear immunofluorescence staining and crispant histology). Parametric tests were performed only where datasets passed a Shapiro-Wilk test for normality. Where possible, tests that do not assume equal standard deviation between groups were performed. All graphs were produced in Prism except the radial histograms, which were created in MATLAB 2025 (Mathworks) using the *polarhistograms* function. All error bars on bar graphs display SEM upper and lower values.

### Graphics and photograph editing

All schematics were produced by the authors. Schematic figure panels were generated in CorelDRAW® V7 vector graphics software. Photograph editing and figure generation were performed in Adobe® Photoshop 2025. Image size and colour levels were adjusted for clarity. All transformations were performed equally over all experimental conditions

### Reporting summary

Further information on research design is available in the Nature Portfolio Reporting Summary linked to this article.

## Supplementary information

Supplemental Information

Description of Additional Supplementary files

Supplementary Data

Supplementary Video 1

Supplementary Video 2

Reporting Summary

Transparent Peer Review file

## Data Availability

The source data for all charts/graphs presented in this manuscript can be found in the supplementary data excel file provided with this paper. Reagents generated are available upon request.
