# Supplementary material for: Overlapping expression and co-operative function of the zebrafish pcdh15 paralogs
Source: Commun Biol. 2026 May 18;9:1048. doi: 10.1038/s42003-026-10289-7 (PMC13443151; doi:10.1038/s42003-026-10289-7)
Supplement: Supplementary file 1 [file 42003_2026_10289_MOESM1_ESM.pdf]

Supplementary Information

Overlapping expression and co-operative function of the zebrafish *pcdh15* paralogs

Paul W. Chrystal[1*], Lisa Kuzmanova[1], Jingpin Liu[1], Alexa Izvorean[1], Ishmael Majeed[1], Jefferson F. Tjoenardi[1], Qian Lin[1], Vincent Tropepe[1*]

[1] Department of Cell & Systems Biology, University of Toronto, Toronto, ON, Canada
[*] Corresponding authors: p.chrystal@utoronto.ca; v.tropepe@utoronto.ca

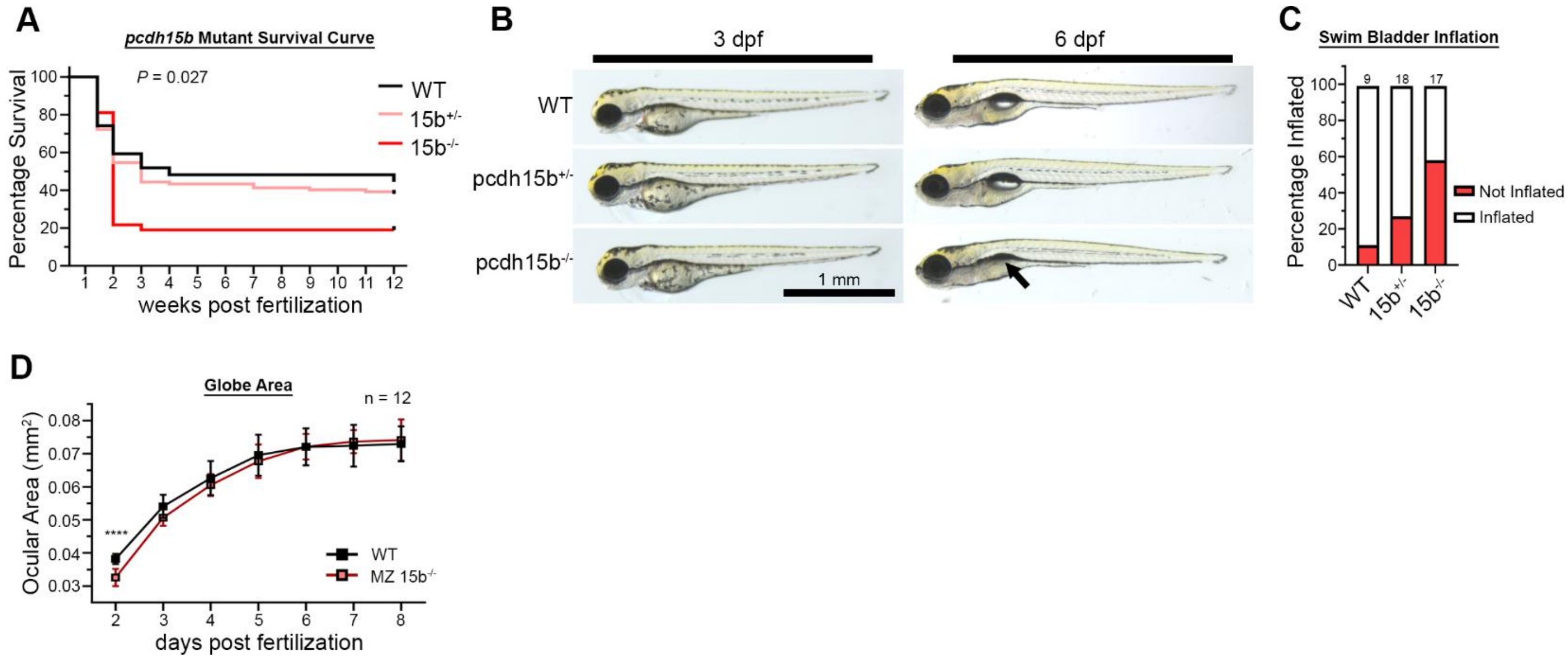

Supplementary Figure S1 – Homozygous *pcdh15b*$^{-/-}$ mutants are viable with mild developmental deformities. A) Survival curve analysis over the first 12 weeks demonstrates a significant decrease in fitness of *pcdh15b*$^{-/-}$ homozygous animals (Kaplan-Meier survival analysis *P* = 0.0266; n-values: WT = 54, *15b*$^{+/-}$ = 97; *15b*$^{-/-}$ = 37). **B)** Assessment via light microscopy (3 and 6 dpf) reveals that *pcdh15b*$^{-/-}$ homozygotes develop without gross abnormalities but fail to inflate the swim bladder (black arrow). **C)** The frequency of swim bladder inflation is inversely proportional to the number of inherited mutant copies (n-values above each bar). **D)** During early development, ocular globe size is reduced in *pcdh15b*$^{-/-}$ homozygotes but this difference is resolved at later stages (*P* = 0.0005, 0.0537, 0.3851, 0.5258, 0.8745, 0.3532, 0.5651; multiple paired t-tests with two-stage step-up FDR correction).

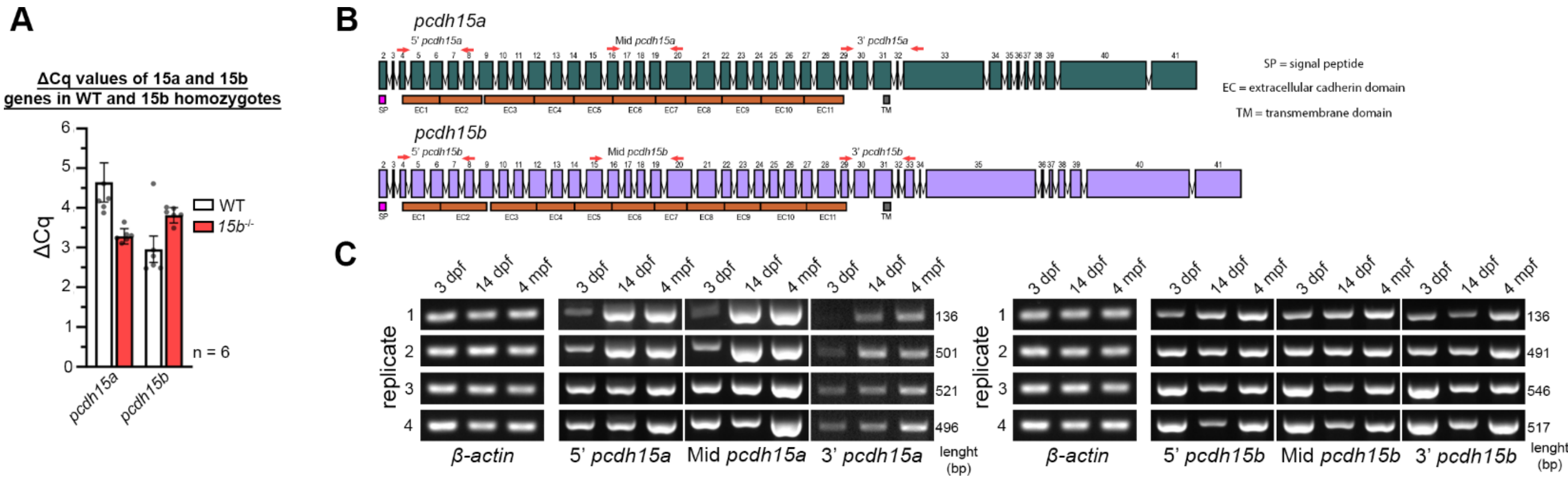

Supplementary Figure S2 – *pcdh15a* and *pcdh15b* expression in the zebrafish retina. A) The ΔCq values for *pcdh15* paralogs in the retina at 8 dpf of WT and *pcdh15b*$^{-/-}$ homozygotes. **B)** Schematic representation of the *pcdh15a* and *pcdh15b* genes. Boxes = exons, lines = introns, arrows = forward and reverse primer binding. The major functional domains are shown below each schematic: SP = signal peptide; EC = extracellular cadherin repeat; TM = transmembrane domain. **C)** Endpoint RT-PCR using three pan-*pcdh15a* and three pan-*pcdh15b* primer pairs demonstrates that both genes are expressed at 3 dpf, 14 dpf, and 4 mpf in the eye.

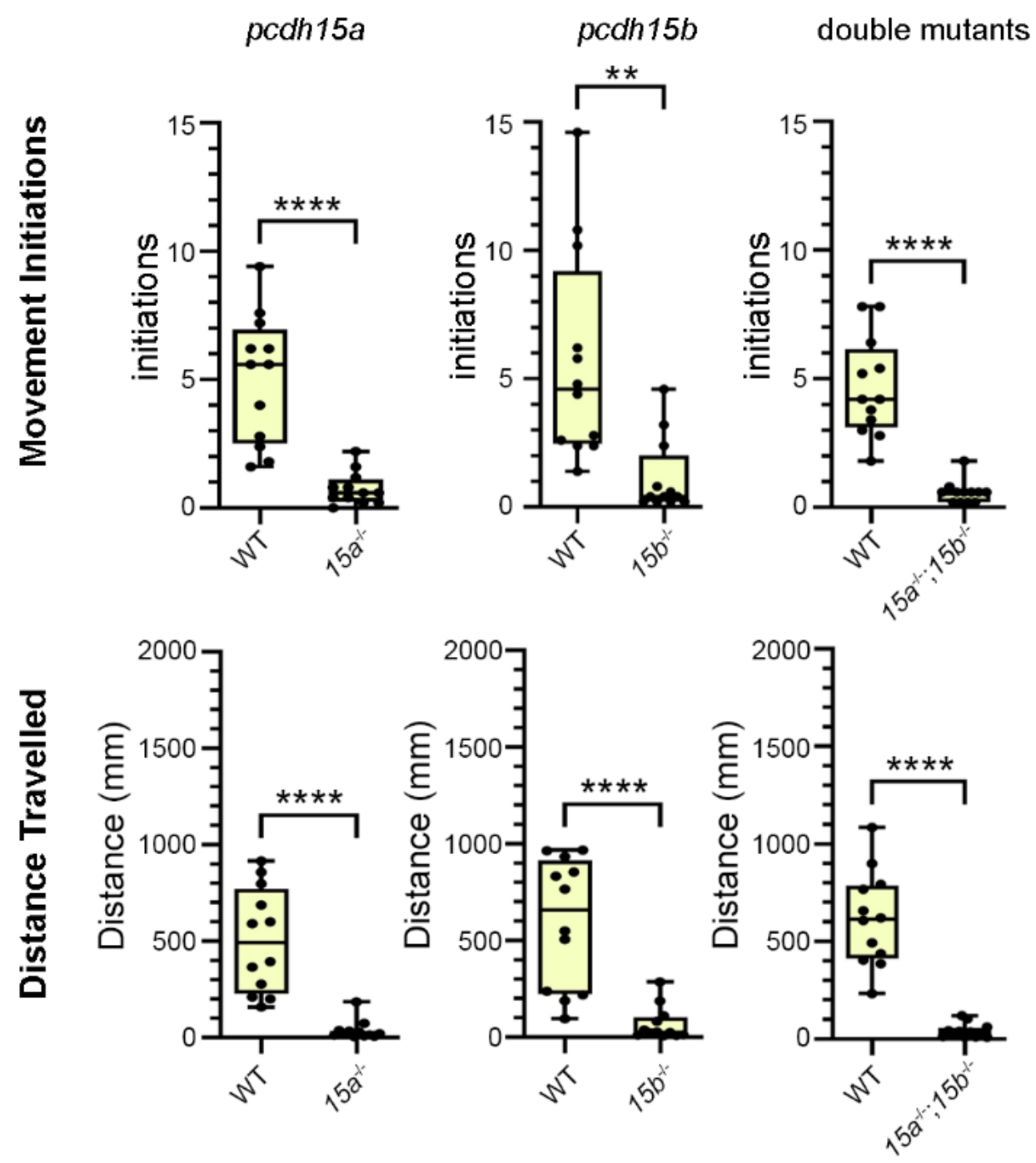

Supplementary Figure S3 – *pcdh15* single and double mutants move less under constant lighting. Locomotor assay performed under constant light conditions. All genotypes tested (*pcdh15a*$^{-/-}$, *pcdh15b*$^{-/-}$, and *pcdh15a*$^{-/-}$;*pcdh15b*$^{-/-}$ double homozygotes) had reduced movement initiations and distance travelled compared to WT controls (Initiations: P = <0.0001 *15a*$^{-/-}$, 0.0029 *15b*$^{-/-}$, <0.0001 *15a*$^{-/-}$;*15b*$^{-/-}$ Welch's t-test. Distance: P = <0.0001 *15a*$^{-/-}$, <0.0001*15b*$^{-/-}$, <0.0001 *15a*$^{-/-}$;*15b*$^{-/-}$unpaired t-test; n-value = 12).

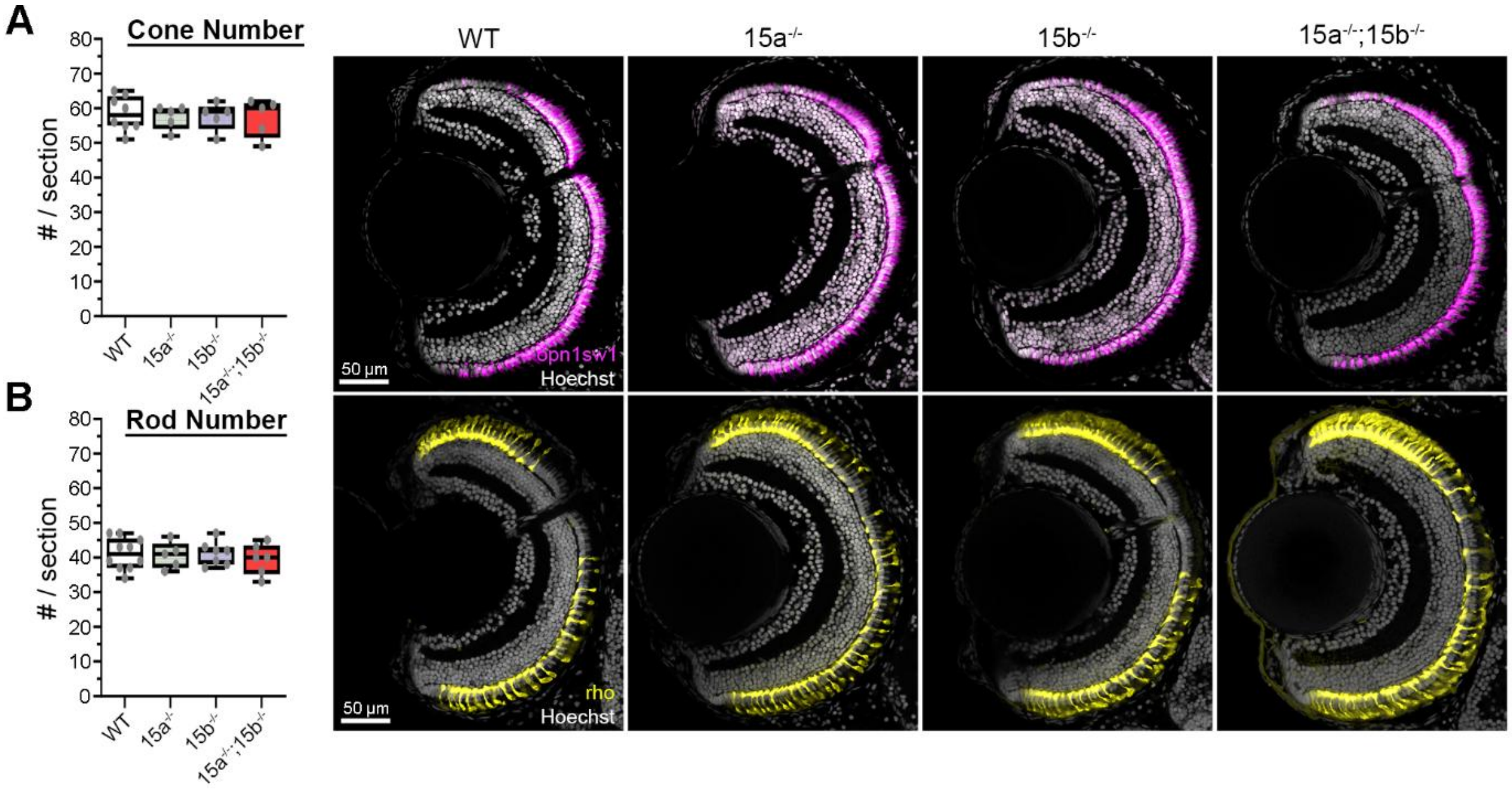

Supplementary Figure S4 – No difference in number of cone or rod cells is detectable in double mutants during development. A) Representative confocal micrograph of each genotype at 8 dpf on the Tg[opn1sw1:EGFP] background and quantification of the number of UV cones per optical section (*P* = 0.9547 Brown-Forsythe and Welch one-way ANOVA, n-value = 8, 5, 5, 5). **B)** Representative micrographs at 8 dpf on the Tg[rho:EGFP] background and quantification of the number of rod cells per optical section (*P* = 0.8709 Brown-Forsythe and Welch one-way ANOVA; n-value = 10, 5, 7, 6).

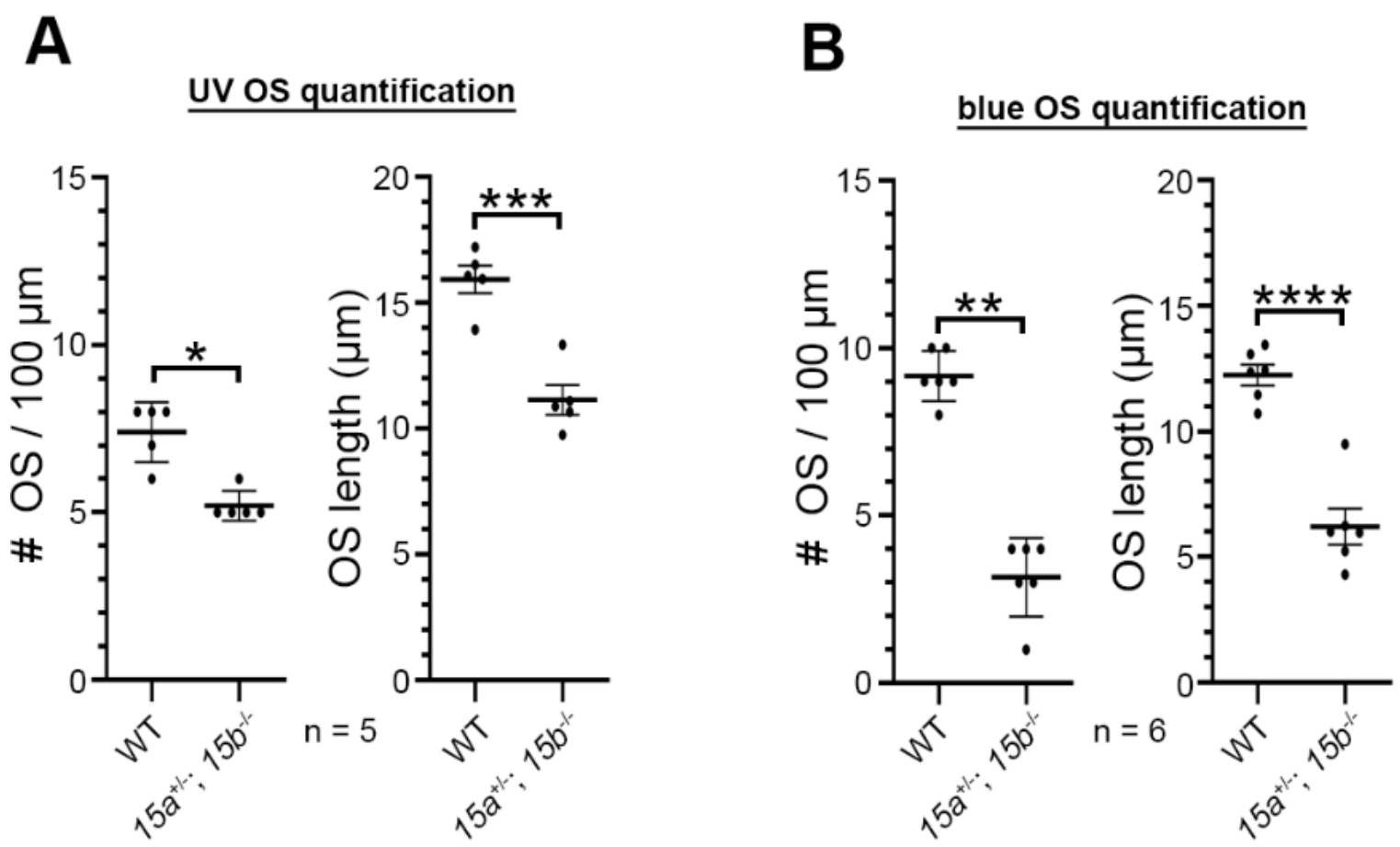

Supplementary Figure S5 – Quantification of cone outer segments in the *pcdh15a*$^{+/-}$; *pcdh15b*$^{-/-}$ mixed zygosity adult retina. A) Following quantification, both the number of UV outer segments (*P* = 0.0159; Mann Whitney test) and length of UV outer segments (*P* = 0.0004; Welch's T-test) was found to be reduced in the central retina of 18 mpf mixed zygosity double mutants. **B)** Quantification of blue cone outer segments also revealed a reduction in number (*P* = 0.0022; Mann Whitney test), and reduced length (*P* <0.0001; Welch's T-test) compared to age-matched WT controls.

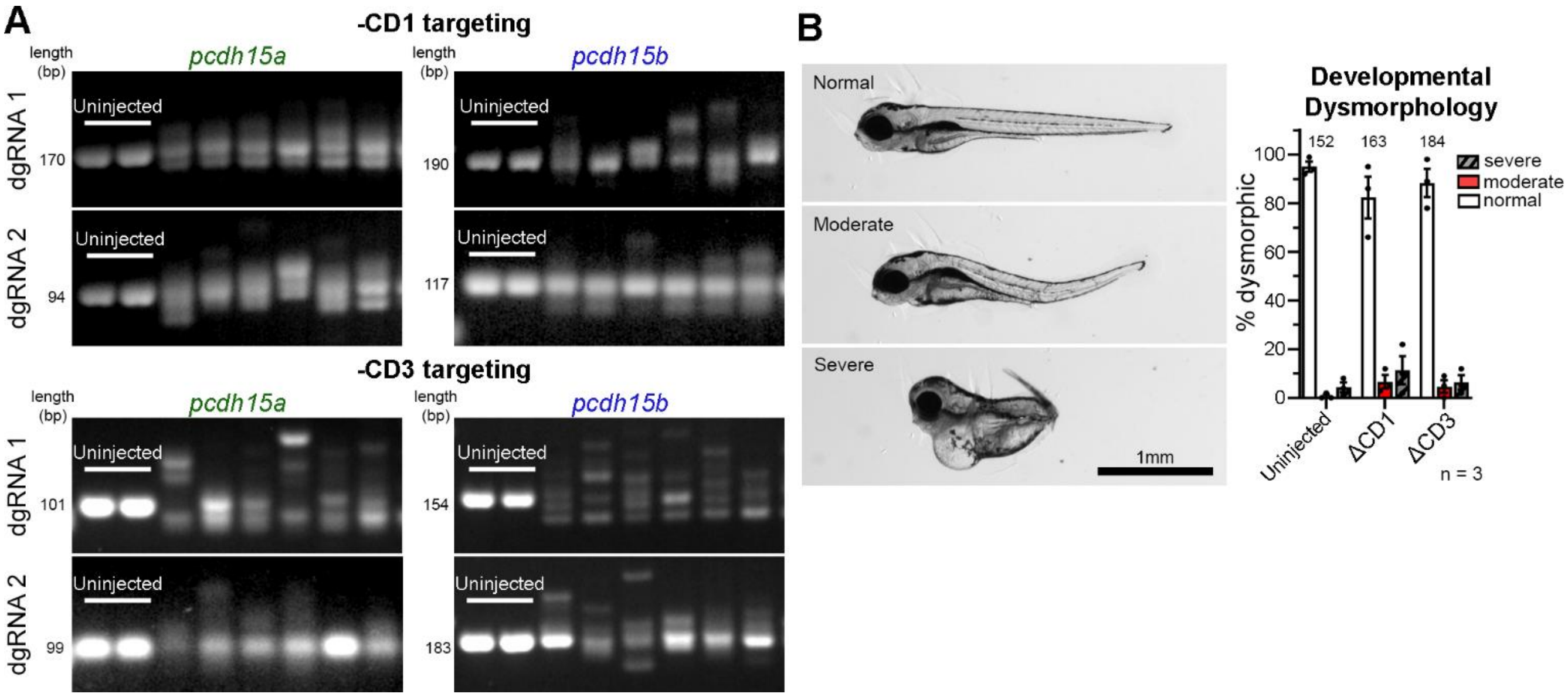

Supplementary Figure S6 – dgRNAs have high cutting efficiency without causing developmental dysmorphology. A) Representative electrophoresis images demonstrating the frequency of indels created by dgRNA injection. The first two lanes of each gel are occupied by reactions from uninjected controls. **B)** Injection of 4 dgRNAs to generate the ΔCD1 knockouts or the ΔCD3 knockouts resulted in <20% of embryos with developmental dysmorphology at 5 dpf (embryos per condition displayed above).

A

AY772391.1 - annotated *pcdh15b* sequence deposited by Seiler et al.

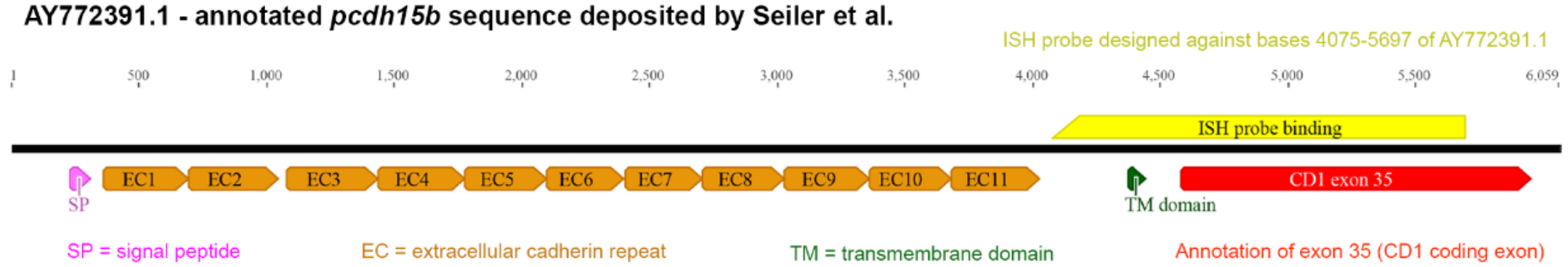

B

Percentage sequence identity matrix

	Probe	CD1	CD2	CD3
Probe	100.0	-	-	-
CD1	99.6	100.0	-	-
CD2	68.9	68.7	100.0	-
CD3	74.1	74.3	82.6	100.0

C

Sequence alignment of Seiler et al. ISH probe Vs *pcdh15b*-CD1, -CD2 and -CD3 (3' of the EC repeats)

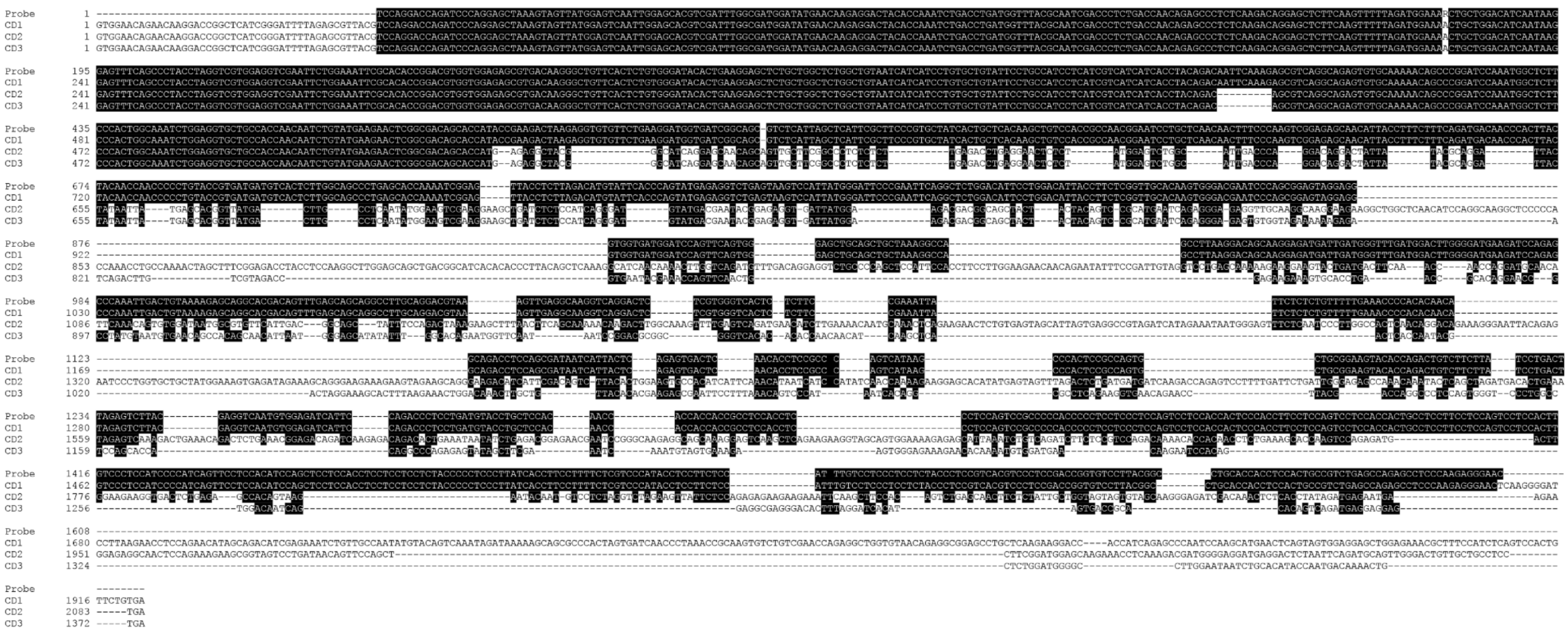

Supplementary Figure S7 – The *in situ* hybridization probe from Seiler et al. 2005 is complementary to the *pcdh15b*-CD1 isoform. A) Schematic representation of the *pcdh15b* nucleotide sequence deposited to NCBI database by Seiler et al. annotated to show where protein functional domains are encoded, and where the *in situ* hybridization probe sequence is located (i.e. predominantly cytoplasmic domain). **B)** The percentage sequence ID matrix of the ISH probe Vs. cytoplasmic regions from *pcdh15b*-CD1 (NM_001012486.1), -CD2 (XM_073917589.1), and -CD3 (XM_073917597.1) transcripts. **C)** Raw alignments of the *in situ* probe Vs *pcdh15b*-CD1, -CD2 and -CD3 sequences. Black highlighting indicates base pair homology.

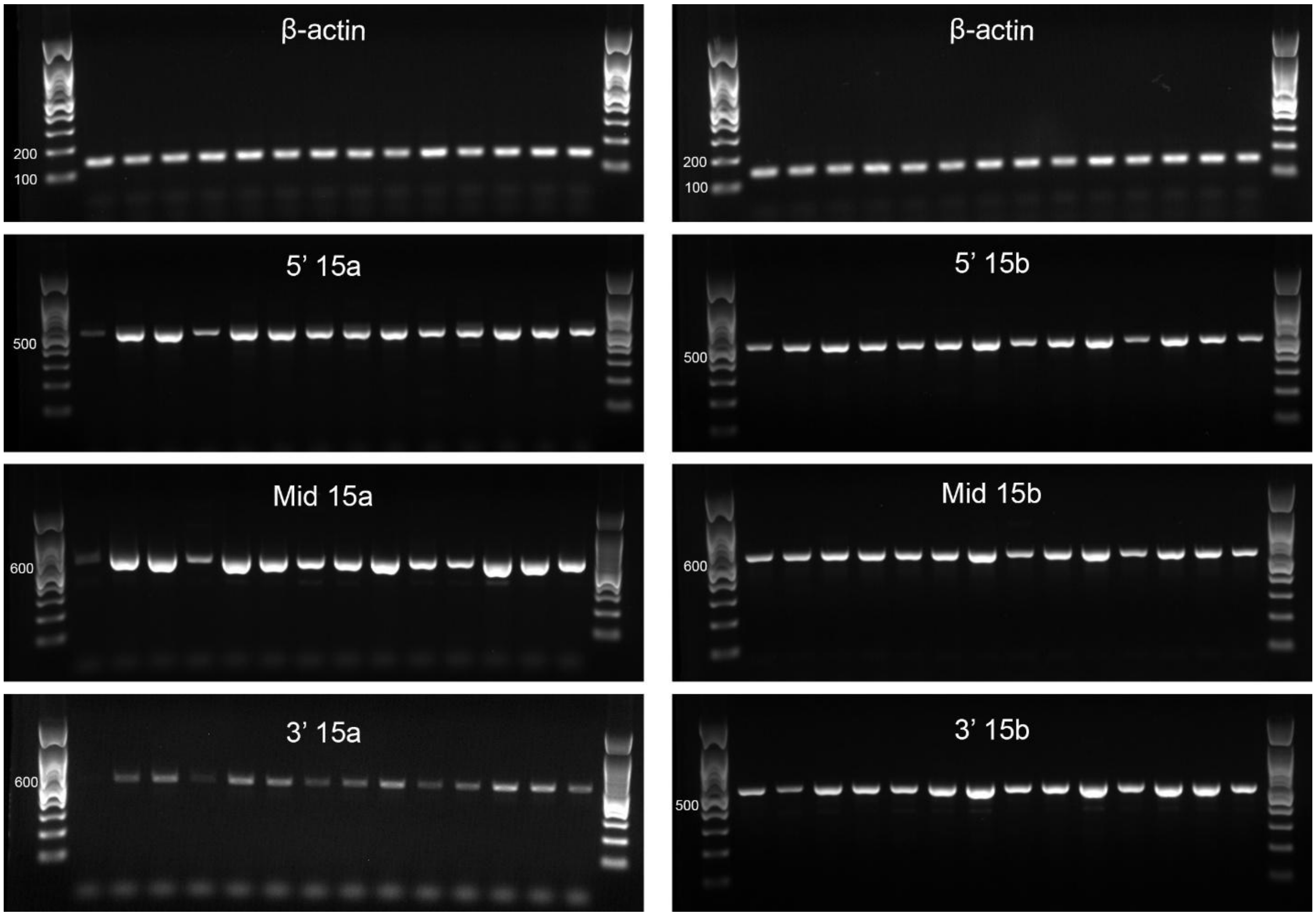

Supplementary Figure S8 Uncropped gel images used to generate figure S2.

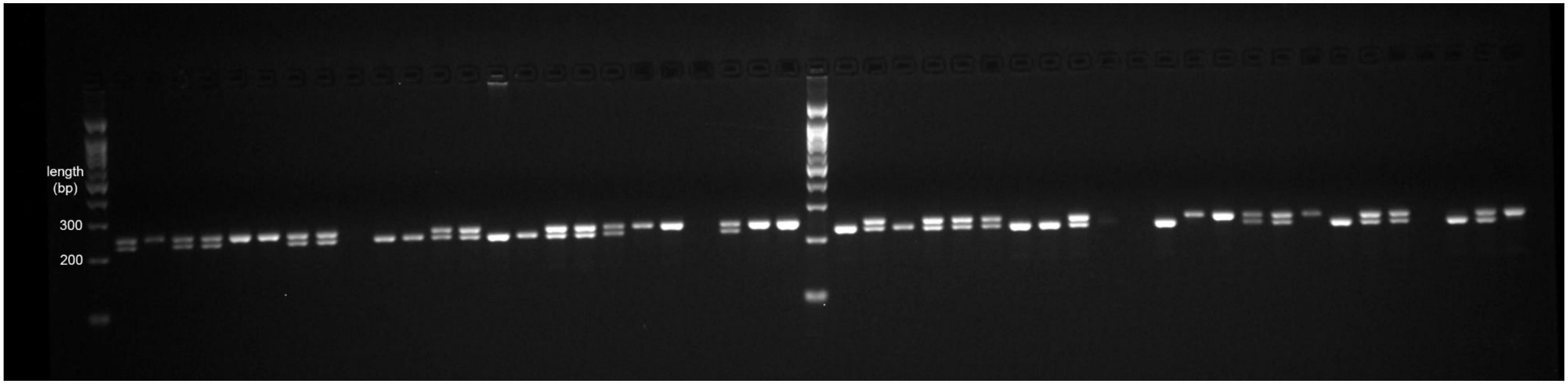

Supplementary Figure S9 - Uncropped gel images used to generate figure 4C.

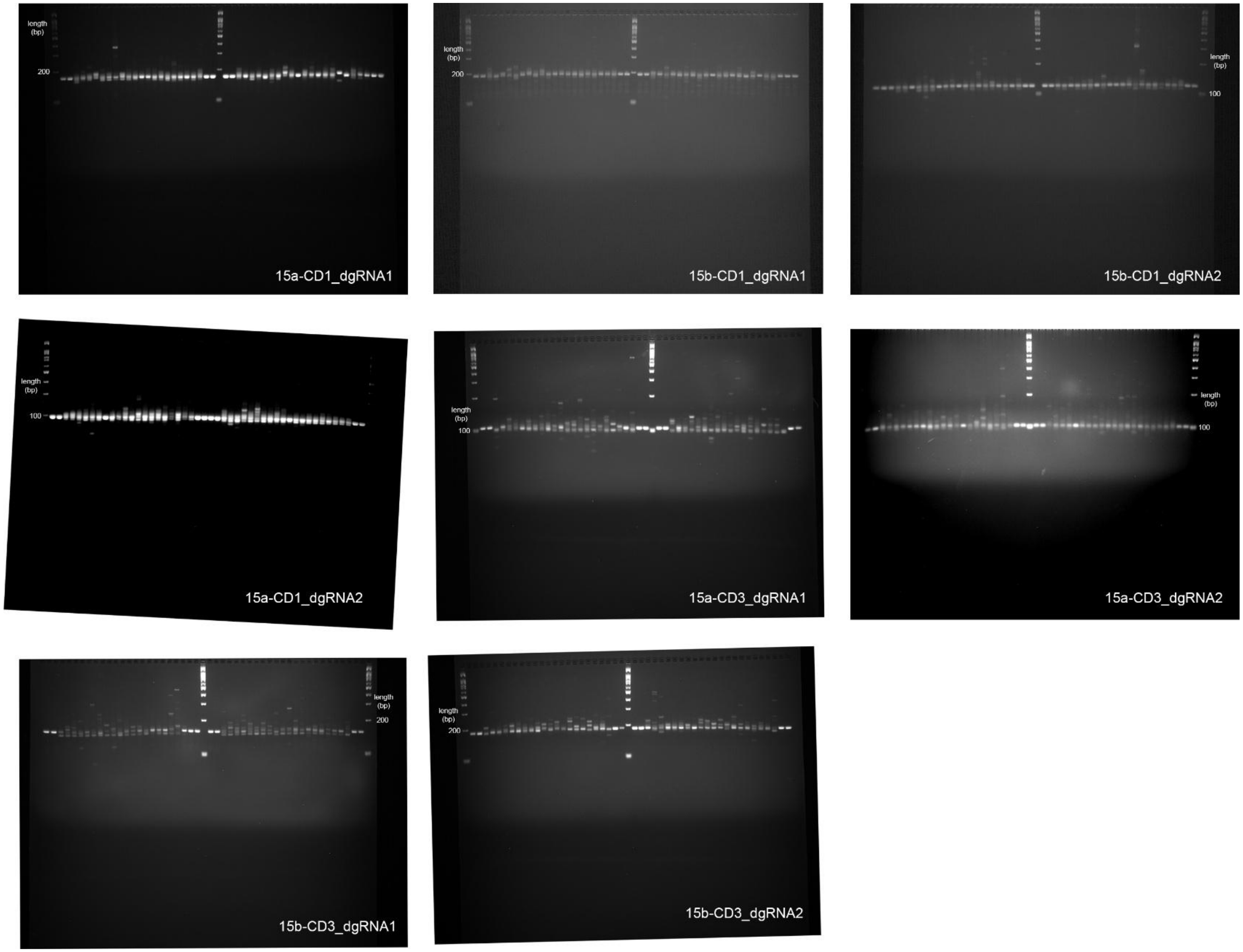

Supplementary Figure S10 – Uncropped gel images used to generate figure S6

Supplementary Tables

Supplementary Table 1 – NCBI catalogued *PCDH15* transcripts *Homo sapiens*

H. sapiens PCDH15 NCBI GeneID: 65217				
**Accession #**	**Transcript Length (bp)**	**Transcript Protein Accession**	**Protein Length (aa)**	**CTD Usage**
NM_001142763.2	6,983	NP_001136235.1	1,962	CD1
NM_001142764.2	6,968	NP_001136236.1	1,957	CD1
NM_033056.4	6,962	NP_149045.3	1,955	CD1
NM_001142766.2	6,953	NP_001136238.1	1,952	CD1
NM_001142768.2	6,902	NP_001136240.1	1,935	CD1
NM_001354404.2	6,923	NP_001341333.1	1,933	CD1
NM_001142773.2	6,893	NP_001136245.1	1,932	CD1
NM_001142767.2	6,842	NP_001136239.1	1,915	CD1
NM_001142765.2	6,755	NP_001136237.1	1,886	CD1
NM_001142769.3	6,330	NP_001136241.1	1,790	CD2
NM_001354411.2	6,309	NP_001341340.1	1,783	CD2
XM_047425664.1	6,048	XP_047281620.1	1,783	CD2
XM_054366621.1	9,216	XP_054222596.1	1,783	CD2
NM_001142770.3	6,365	NP_001136242.1	1,539	CD2
NM_001384140.1	9,366	NP_001371069.1	1,740	CD3
NM_001354429.2	9,300	NP_001341358.1	1,718	CD3
NM_001142771.2	9,192	NP_001136243.1	1,682	CD3
NM_001142772.2	9,177	NP_001136244.1	1,677	CD3
NM_001354420.2	9,171	NP_001341349.1	1,675	CD3
XM_047425663.1	9,162	XP_047281619.1	1,672	CD3
XM_054366620.1	9,162	XP_054222595.1	1,672	CD3
NM_001354430.2	4,280	NP_001341359.1	961	N/A

Supplementary Table 2 – NCBI catalogued *pcdh15* transcripts *Danio rerio*

D. rerio *pcdh15a* NCBI GeneID: 368476				
**Accession #**	**Transcript Length (bp)**	**Transcript Protein Accession**	**Protein Length (aa)**	**CTD Usage**
NM_001012482.1	6,521	NP_001012500.1	1,796	CD1
XM_073919397.1	8,172	XP_073775498.1	2,123	CD2
XM_017358710.4	6,408	XP_017214199.1	1,726	CD3
D. rerio pcdh15b NCBI GeneID: 503521				
**Accession #**	**Transcript Length (bp)**	**Transcript Protein Accession**	**Protein Length (aa)**	**CTD Usage**
NM_001012486.1	6,059	NP_001012504.1	1,907	CD1
XM_073917589.1	8,092	XP_073773690.1	1,961	CD2
XM_073917590.1	8,112	XP_073773691.1	1,961	CD2
XM_073917591.1	8,236	XP_073773692.1	1,961	CD2
XM_073917588.1	7,589	XP_073773689.1	1,961	CD2
XM_073917593.1	8,077	XP_073773694.1	1,956	CD2
XM_073917594.1	8,097	XP_073773695.1	1,956	CD2
XM_073917592.1	7,574	XP_073773693.1	1,956	CD2
XM_073917595.1	7,550	XP_073773696.1	1,948	CD2
XM_073917596.1	8,038	XP_073773697.1	1,943	CD2
XM_073917597.1	6,285	XP_073773698.1	1,724	CD3
XM_073917598.1	6,773	XP_073773699.1	1,719	CD3
XM_073917599.1	6,897	XP_073773700.1	1,719	CD3
XM_073917600.1	6,917	XP_073773701.1	1,719	CD3
XM_073917601.1	6,246	XP_073773702.1	1,711	CD3
XM_073917603.1	6,734	XP_073773704.1	1,706	CD3
XM_073917605.1	6,780	XP_073773706.1	1,706	CD3
XM_073917604.1	6,754	XP_073773705.1	1,706	CD3
XM_073917606.1	6,100	XP_073773707.1	1,706	CD3
XM_073917602.1	6,231	XP_073773703.1	1,706	CD3
XM_073917607.1	5,538	XP_073773708.1	1,517	N/A
XM_073917608.1	6,705	XP_073773709.1	1,412	N/A

Non-coding transcripts omitted for conciseness

Supplementary Table 3 – Zebrafish key resources

Zebrafish stains

Name	Source	Zfin ID
AB	ZIRC	ZDB-GENO-960809-7
Tg(-3.7rho:EGFP)kj2Tg	Hamaoka, 2002 PMID: 12395387	ZDB-ALT-060830-4
Tg(-5.5opn1sw1:EGFP)kj9	Takechi, 2003 PMID: 14550552	ZDB-ALT-080227-1
pcdh15b^{uot15}	Miles, 2021 PMID: 34668518	ZDB-ALT-211130-2

Antibodies / counterstains

Name	Source	Product ID
Anti-Zebrafish UV Opsin	Kerafast	EJH013
Anti-Zebrafish Blue Opsin	Kerafast	EJH012
Anti-cleaved caspase-3	Cell Signaling Technology	#9661
Anti-GFP	Invitrogen	A-11122
Anti-PCDH15	Abcam	ab202560
Anti-COX IV antibody	Abcam	ab33985
Anti-Tubulin, Acetylated	Millipore Sigma	T6793
488 Alexa Fluor™ goat anti-rabbit	ThermoFisher Scientific	A-11008
488 Alexa Fluor™ goat anti-mouse	ThermoFisher Scientific	A-11001
555 Alexa Fluor™ goat anti-rabbit	ThermoFisher Scientific	A-21428
555 Alexa Fluor™ goat anti-mouse	ThermoFisher Scientific	A-21422
555 Alexa Fluor™ Wheat Germ Agglutinin	ThermoFisher Scientific	W32464
647 Alexa Fluor™ Phalloidin	Cell Signaling Technology	8940
Hoechst 33342	ThermoFisher Scientific	H3570

Primers

Name	Sequence	Source / Citation
pcdh15a_genotyping_Fwd	GTCCGTCTCCCTTTATGCACCT	IDT
pcdh15a_genotyping_Rev	TCGGTCCAGCACACGTCC	IDT
pcdh15b_genotyping_Fwd	TAACGCCTGTCGGAACCA	IDT
pcdh15b_genotyping_Rev	ATTCAATCTGTCCATTAGGACCA	IDT
B-actin_qPCR_Fwd	CGGACAGGTCATCACCATTG	PMID: 23523635
B-actin_qPCR_Rev	GATGTCGACGTCACACTTCA	
lsm12b_qPCR_Fwd	AGGCAAAGAGGGAAGTGCTC	IDT
lsm12b_qPCR_Rev	TGAACGCTGCATCGTCTTCT	IDT
mob4_qPCR_Fwd	GCTGCATGTCTTCTCAACAGC	IDT
mob4_qPCR_Rev	TCCTATAGATGCGACGGCAA	IDT
pan -pcdh15a_ qPCR_Fwd	AGAGCCATCTCCAGACAAGAG	IDT
pan-pcdh15a_qPCR_Rev	CTCCAGAATACGCCCACCCT	IDT
pan -pcdh15b_ qPCR_Fwd	ATCGACCCTCTGACCAACAG	IDT
pan-pcdh15b_qPCR_Rev	GAATTCGACCTCCACGACCT	IDT
pcdh15a_CD1_ qPCR_Fwd	CTTGGACTACAAAGACGCAGAAG	IDT
pcdh15a_CD1_ qPCR_Rev	TCCAGTGGTCTGTTTGTGAGC	IDT

pcdh15a_CD2_ qPCR_Fwd	AGGAGGCTGATCACTCCGAT	IDT
pcdh15a_CD2_ qPCR_Rev	CTCTTCCCCTACCCTCTGGG	IDT
pcdh15a_CD3_ qPCR_Fwd	TGGAGGATGAAGGTTATTACTATGG	IDT
pcdh15a_CD3_ qPCR_Rev	CTCTCCTGTTGAAGTCGCG	IDT
pcdh15b_CD1_ qPCR_Fwd	CCAACAATCTGTATGAAGAACTCG	IDT
pcdh15b_CD1_ qPCR_Rev	CAGTGATAGCACGGGAAGC	IDT
pcdh15b_CD2_ qPCR_Fwd	TACAGTCCGCATGAATCAGAG	IDT
pcdh15b_CD2_ qPCR_Rev	AAAGCTAGTTTTGGCAGGTTTGG	IDT
pcdh15b_CD3_ qPCR_Fwd	GAGAGGTGATTATGGAAGACG	IDT
pcdh15b_CD3_ qPCR_Rev	CGTATTCACGGTCTACGAC	IDT
5'_pcdh15a_Fwd	TCACCATTGATGAGGAAAGTCC	IDT
5'_pcdh15a_Rev	ACAGCGTCCCAGCAATCC	IDT
Mid_pcdh15a_Fwd	CTACGGTTTACATAGAAGTTCTGC	IDT
Mid_pcdh15a_Rev	GAAGATGCTGCCTGTGGAG	IDT
3'_pcdh15a_Fwd	TTCCTCCTACAGTGGTGGAG	IDT
3'_pcdh15a_Rev	GGAAGTGCCATCTGGATACG	IDT
5'_pcdh15b_Fwd	CATCGTAGCCATCGATGAGC	IDT
5'_pcdh15b_Rev	TGTCGAATGTCCTGTTAGTGG	IDT
Mid_pcdh15b_Fwd	TGCTGCCTAGCGTCAACC	IDT
Mid_pcdh15b_Rev	TAATGCCCAGGTCCAGATCG	IDT
3'_pcdh15b_Fwd	GTGGTCGTATCCAACGTGC	IDT
3'_pcdh15b_Rev	CAGTGGGAAGAGCCATTTGG	IDT
15a-CD1_guide1_Fwd	CAGACCACTGGAAACGGACA	IDT
15a-CD1_guide1_Rev	GCTTGTTGGAGAAGGTGGGT	IDT
15a-CD1_guide2_Fwd	GGCTTTGGCTGATCGAACCC	IDT
15a-CD1_guide2_Rev	GTGGAGGAGGAGTCTCACCAT	IDT
15a-CD3_guide1_Fwd	CTATCCACCTTATGGCAGCCG	IDT
15a-CD3_guide1_Rev	CTGGGCCACCATAGTAATAACC	IDT
15a-CD3_guide2_Fwd	GCAATCGCATACCCAACGTC	IDT
15a-CD3_guide2_Rev	CATAAGGGGGCCTTCGTGTT	IDT
15b-CD1_guide1_Fwd	CTTTCCCAAGTCGGAGAGCA	IDT
15b-CD1_guide1_Rev	GCCTGAATTCGGGAATCCCAT	IDT
15b-CD1_guide2_Fwd	CACCTCCGCCCAGTCATAAG	IDT
15b-CD1_guide2_Rev	CAGGAGGGTCTGGAATGATCTC	IDT
15b-CD3_guide1_Fwd	TCAGACTTGTCGTAGACCGTG	IDT
15b-CD3_guide1_Rev	CGCGTCCGGATTATTGAACC	IDT
15b-CD3_guide2_Fwd	GTTCAATAATCCGGACGCGG	IDT
15b-CD3_guide2_Rev	GCCTGGTCGTAAGGTTCTGT	IDT

gRNAs

Name	Sequence	Source
pcdh15a_crRNA_1	GUUGAGGAACAGUCUCUGCA	IDT
pcdh15a_crRNA_2	GCUGGACAACUAUGACUAUU	IDT
15a-CD1_gRNA_3	AACCUAACUUGGAGGUCCGA	IDT
15a-CD1_gRNA_2	GAUCCUGACGACCCACUGCU	IDT
15a-CD3_gRNA_1	UCUCCAUACUCAUCAUACAU	IDT
15a-CD3_gRNA_3	UCAGUAUACCUAGCCCAAAA	IDT
15b-CD1_gRNA_1	GGGGGUUGGUUGUAGUAAGU	IDT
15b-CD1_gRNA_2	UCCUGACUUAGAGUCUUACG	IDT

15b-CD3_gRNA_1	AAAGUGCACCUGAACCGCAC	IDT
15b-CD3_gRNA_2	UCAACUCUCCAAUACGACUA	IDT

Chemicals

**Name**	**Source**	**Catalogue ID#**
PFA	Sigma-Aldrich	P6148
O.C.T. compound	Sakura	4583
BSA	BioShop Canada	ALB001
normal goat serum	Cell Signaling Technology	5425
ProLong™ Gold	Invitrogen	P36930
Sodium citrate	Fsher Scientific	S-279
Tween-20	BioShop Canada	TWN510
Triton X-100	BioShop Canada	TRX777
T4 DNA Polymerase	New England Biolabs	M0203S
SP6 MEGAscript Kit	Thermofisher Scientific	AM1330
EnGen Spy Cas9 NLS	New England Biolabs	M0646M
Tungsten wire	Fine Science Tools	10130-05
Eugenol	Sigma-Aldrich	W246700
GoTaq G2	Promega	M7848
dNTPs	Sigma-Aldrich	GE28-4065
TRIzol™ Reagent	Thermofisher Scientific	15596026
SuperScript™ IV Reverse Transcriptase	Thermofisher Scientific	18090010
GoTaq® G2 DNA Polymerase	Promega	M7848
LightCycler® 480 SYBR Green I Master	Roche	04887352001
LMP agarose	IBI Scientific	IB70051

Supplementary Table 4 – *P*-values of locomotor activity assay

Genotype	Initiations	Distance
WT	0.0183	<0.0001
pcdh15a$^{-/-}$	0.9864	0.9328
WT	0.0183	0.0001
pcdh15b$^{-/-}$	0.9864	0.9950
WT	0.0012	0.0010
pcdh15a$^{-/-}$; *pcdh15b*$^{-/-}$	0.9962	0.9708

P-values corresponding to Fig. 6B
