# Supplementary material for: Overlapping expression and co-operative function of the zebrafish pcdh15 paralogs
Source: Commun Biol. 2026 May 18;9:1048. doi: 10.1038/s42003-026-10289-7 (PMC13443151; doi:10.1038/s42003-026-10289-7)
Supplement: Supplementary file 2 [file 42003_2026_10289_MOESM6_ESM.pdf]

nature portfolio

Corresponding author(s): Paul W. Chrystal

Last updated by author(s): Apr 16, 2026

Reporting Summary

Nature Portfolio wishes to improve the reproducibility of the work that we publish. This form provides structure for consistency and transparency in reporting. For further information on Nature Portfolio policies, see our Editorial Policies and the Editorial Policy Checklist.

Statistics

For all statistical analyses, confirm that the following items are present in the figure legend, table legend, main text, or Methods section.

n/a	Confirmed	
☐	☒	The exact sample size (*n*) for each experimental group/condition, given as a discrete number and unit of measurement
☒	☐	A statement on whether measurements were taken from distinct samples or whether the same sample was measured repeatedly
☐	☒	The statistical test(s) used AND whether they are one- or two-sided<br>*Only common tests should be described solely by name; describe more complex techniques in the Methods section.*
☒	☐	A description of all covariates tested
☐	☒	A description of any assumptions or corrections, such as tests of normality and adjustment for multiple comparisons
☐	☒	A full description of the statistical parameters including central tendency (e.g. means) or other basic estimates (e.g. regression coefficient) AND variation (e.g. standard deviation) or associated estimates of uncertainty (e.g. confidence intervals)
☐	☒	For null hypothesis testing, the test statistic (e.g. *F*, *t*, *r*) with confidence intervals, effect sizes, degrees of freedom and *P* value noted<br>*Give P values as exact values whenever suitable.*
☒	☐	For Bayesian analysis, information on the choice of priors and Markov chain Monte Carlo settings
☒	☐	For hierarchical and complex designs, identification of the appropriate level for tests and full reporting of outcomes
☒	☐	Estimates of effect sizes (e.g. Cohen's *d*, Pearson's *r*), indicating how they were calculated

Our web collection on statistics for biologists contains articles on many of the points above.

Software and code

Policy information about availability of computer code

Data collection	N/A
Data analysis	MATLAB 2025 (Mathworks),<br>GraphPad Prism V10,<br>CorelDRAW® V7,<br>Adobe® Photoshop 2025,<br>DeepLabCut,<br>FIMTrack.

For manuscripts utilizing custom algorithms or software that are central to the research but not yet described in published literature, software must be made available to editors and reviewers. We strongly encourage code deposition in a community repository (e.g. GitHub). See the Nature Portfolio guidelines for submitting code & software for further information.

Data

Policy information about availability of data

All manuscripts must include a data availability statement. This statement should provide the following information, where applicable:

- Accession codes, unique identifiers, or web links for publicly available datasets
- A description of any restrictions on data availability
- For clinical datasets or third party data, please ensure that the statement adheres to our policy

All accession numbers for nucleotides used in this study are listed in supplementary tables 1 and 2. The nucleotide sequence used in Supp. Fig. 6 is AY772391.1. All data generated during this study will be included in this article, its Supplementary file, and the Source Data file provided with this paper.

Research involving human participants, their data, or biological material

Policy information about studies with human participants or human data. See also policy information about sex, gender (identity/presentation), and sexual orientation and race, ethnicity and racism.

Reporting on sex and gender	N/A
Reporting on race, ethnicity, or other socially relevant groupings	N/A
Population characteristics	N/A
Recruitment	N/A
Ethics oversight	N/A

Note that full information on the approval of the study protocol must also be provided in the manuscript.

Field-specific reporting

Please select the one below that is the best fit for your research. If you are not sure, read the appropriate sections before making your selection.

☒ Life sciences ☐ Behavioural & social sciences ☐ Ecological, evolutionary & environmental sciences

For a reference copy of the document with all sections, see nature.com/documents/nr-reporting-summary-flat.pdf

Life sciences study design

All studies must disclose on these points even when the disclosure is negative.

Sample size	For all experiments, biological replicates were defined as individual animals unless otherwise stated. Adult histology sample size was ≥ 3 due to time and resource constraints. Larval histology sample size was ≥ 6. For behavioral tests (OKR, locomotor tests) which are inherently more variable, sample size was ≥ 12 per condition. qRT-PCR analysis was performed on pooled cDNA samples with sample size 6. Cutting efficiency analysis during crispant generation had a sample size of 40 to determine spread of the data in addition to mean efficiency.
Data exclusions	Samples were excluded from all conditions of the OKR analysis if larvae became unresponsive during the 180 second recording.
Replication	Histological findings were repeated using different tissue and with immunohistochemistry performed on separate days.
Randomization	N/A
Blinding	All samples were double blinded prior to image analysis. For behavioral analysis, genotype of individuals was determined only after samples were assayed.

Reporting for specific materials, systems and methods

We require information from authors about some types of materials, experimental systems and methods used in many studies. Here, indicate whether each material, system or method listed is relevant to your study. If you are not sure if a list item applies to your research, read the appropriate section before selecting a response.

Materials & experimental systems

n/a	Involved in the study
☐	☒ Antibodies
☒	☐ Eukaryotic cell lines
☒	☐ Palaeontology and archaeology
☐	☒ Animals and other organisms
☒	☐ Clinical data
☒	☐ Dual use research of concern
☒	☐ Plants

Methods

n/a	Involved in the study
☒	☐ ChIP-seq
☒	☐ Flow cytometry
☒	☐ MRI-based neuroimaging

Antibodies

Antibodies used

Anti-Zebrafish UV Opsin Kerafast EJH013
Anti-Zebrafish Blue Opsin Kerafast EJH012
Anti-cleaved caspase-3 Cell Signaling Technology #9661
Anti-GFP Invitrogen A-11122
Anti-PCDH15 Abcam ab202560
Anti-COX IV antibody Abcam ab33985
Anti-Tubulin, Acetylated Millipore Sigma T6793
488 Alexa Fluor™ goat anti-rabbit ThermoFisher Scientific
A-11008

488 Alexa Fluor™ goat anti-mouse ThermoFisher Scientific
A-11001
555 Alexa Fluor™ goat anti-rabbit ThermoFisher Scientific
A-21428
555 Alexa Fluor™ goat anti-mouse ThermoFisher Scientific
A-21422
555 Alexa Fluor™ Wheat Germ Agglutinin ThermoFisher Scientific W32464
647 Alexa Fluor™ Phalloidin Cell Signaling Technology 8940
Hoechst 33342 ThermoFisher Scientific H3570

Validation

The only novel antibody not previously validated is the Anti-PCDH15 (Abcam ab202560). In this manuscript we validated the antibody by demonstrating that the fluorescent signal detected by this antibody via immunohistochemistry is abolished when both zebrafish pcdh15 paralogs are mutated.

Animals and other research organisms

Policy information about studies involving animals; ARRIVE guidelines recommended for reporting animal research, and Sex and Gender in Research

Laboratory animals

Zebrafish (Danio rerio), AB strain. Experiments performed at 8 days post fertilization (dpf) or 18 months post fertilization (histology), 2 dpf (FM 1-43), 3 dpf (ear immunos), 6 dpf (behavioural testing), 5 dpf (Crispant cutting efficiency).

Wild animals

N/A

Reporting on sex

Zebrafish do not possess sex chromosomes and are not sexually dimorphic until 4 mpf. Sex was therefore not possible to determine for these experiments. For the qRT-PCR analysis we analyzed pcdh15a and pcdh15b expression between male and female samples and found no significant difference in expression.

Field-collected samples

N/A

Ethics oversight

All experiments were performed in accordance with the regulations for animal experimentation established by the Canadian Council on Animal Care and approved by the University of Toronto Animal Care Committee.

Note that full information on the approval of the study protocol must also be provided in the manuscript.

Plants

Seed stocks	N/A
Novel plant genotypes	N/A
Authentication	N/A
