# Supplementary material for: Overlapping expression and co-operative function of the zebrafish pcdh15 paralogs
Source: Commun Biol. 2026 May 18;9:1048. doi: 10.1038/s42003-026-10289-7 (PMC13443151; doi:10.1038/s42003-026-10289-7)
Supplement: Supplementary file 3 [file 42003_2026_10289_MOESM7_ESM.pdf]

nature portfolio

Peer Review File

Overlapping expression and co-operative function of the zebrafish pcdh15 paralogs

Corresponding Author: Dr Paul Chrystal

This file contains all reviewer reports in order by version, followed by all author rebuttals in order by version.

Version 0:

Reviewer comments:

Reviewer #1

(Remarks to the Author)
The manuscript from Chrystal and co-authors entitled “Overlapping expression and co-operative function of the zebrafish pcdh15 paralogs-the case for generating doble mutants when modelling human disease” is very nicely crafted. The authors provide scholarly science to clarify the function of the two pch15 paralogs in zebrafish and appropriately and directly correct long-standing errors in the literature about the claim of distinctly different nonoverlapping functions of pcdh15a and pcdh15b paralogs reported by prominent scientists. This study is robust important science that corrects the literature and adds considerable new data about the functions of pcdh15 paralogs in zebrafish.

There are no major concerns detected in the manuscript such as absence of important control data or essential missing data. There are remaining questions for future studies. However, there are several recommendations listed below for improving the manuscript starting with the title.

1. The title of the manuscript is too long. Consider “Overlapping expression and co-operative function of the zebrafish pcdh15 paralogs”. The case for studying double mutants has been obvious starting with studies of Drosophila melanogaster a century ago. The point is certainly worth emphasizing again in the abstract and the body of manuscript.
2. Abstract, line 15, consider adding a phrase describing what is meant by “Transcriptional adaptation”. If there is insufficient room in the abstract, define TA the first time used rather than waiting for a definition of TA in the body of the manuscript on line 136.
3. Introduction, line 59, Name the three CD1, CD2 and CD3 at the end of this sentence when first mentioned and provide a reference for the three cytoplasmic domains when CD1, CD2 and CD3 were first reported in mouse.
4. The authors are correct that Pcdh15 mouse models fail to recapitulate the more severe human RP phenotype resulting in progressive loss of vision. However, careful examination of vision physiology of a Pcdh15 mouse model does have abnormalities although not nearly as devesatating as in human with the same truncating variant of PCDH15 (Sethna et al 2021, eLife).
5. Introduction, line85, “dichotomic expression pattern” is pedantic phraseology?
6. Results, line 202, “bathing” for how long?
7. Line 289, what criterion was used to claim nuclei are pyknotic?
8. Lines 301 to 322. There are several terms used without criteria stated including “displaced”, “disordered”, “irregular”, and “disorganized” that would be better science if vague words were associated with some specific detailed data or quantification mentioned in the text.
9. Figure 8 Panel F and G, consider including plots comparing UV OS quantification and blue OS quantification as shown in figure 2 panel D and F. This would strengthen statements on effect of mixed zygosity of pcdh15.
10. Line 319, consider moving the sentence on rod cell susceptibility earlier in the Results section where pcdh15b-/- showing mild outer segment dysmorphology is discussed.
11. Consider moving the section beginning at line 323 titled “Isoform-specific roles for pcdh15” to the beginning of the Results section. The Results section would end with a better transition to the Discussion section with the last sentence of the Results “Taken together, these data reveal…..”.
12. Discussion, As mentioned by the authors, on one side of a tip link are two protocadhein 15 monomers in handshake with two monomers of cadherin 23 as shown in panel B of figure 1. Not mentioned is that during development and when tip links are broken, protocadherin 15 forms tip links on both side without cadherin23. Subsequently, cadherin 23 replaces protocadherin on the upside of the tip link. See Indzhykulian et al., PLOS Biology. For the future, is this phenomenon

reported for mouse present in zebra fish?
13. Materials and Methods, line 455, add details of the fixation not just 4% paraformaldehyde.
14. Is it Spurr epoxy resin not Spurr's?
15. Most of the figures have a font size that is too small even though there is plenty of room to increase the font size.
16. Figure 1, panel A, the CD1, CD2 and CD3 are labelled "Cytoplasmic" but the key calls them C-terminal domain. Both are correct but consider using one or the other but not both in the same figure panel.
17. Figure 1 legend, "large gene" is meaningless and not very scientific. Provide some numbers either the number of exons and or the number of base pairs.
18. Figure 1 panel A, in the table add similarity percents to identity percents.
19. Figure 1, panel B, the MET channel is on the side of a stereocilium not on the top as drawn.
20. Supplementary figure S1, panel B, does the heterozygote fail to inflate the swim bladder? Why is there a black arrow pointing to the swim bladder for heterozygotes?
21. Figure 2, legend, Indicate why the Welch t-test was used. Is this because of unequal variances?
22. Figure 2 Panel D, F, H, consider including the count of samples "n" for consistency between other figures and include significance bars. Include counts for figures 5, 6, S3, S4, 7, 8 and 9.
23. Figure 9 Panel B, SB inflation coloring is confusing with inflated and not inflated, inconsistent with previous SB inflation shown in Supplementary Figure S1 Panel C. Also, n=3 representing clutches in this figure is unclear. Consider providing the number of fish instead.
24. Figure 9 Panel D shows splayed stereocilia and attached kinocilium, which the authors claim indicates a CD3 role in mechanosensation. Are there any comparisons that could be made to figure 5 panel F which shows pcdh15a-/- and pcdh15b-/- double mutant showing splayed stereocilia and detached kinocilium?

Reviewer #2

(Remarks to the Author)
Chrystal and colleagues present a very nice manuscript that studies the effects of inactivation of zebrafish genes which are duplicated in this species alone. In this case, they study orthologs of PCDH15 (protocadherin 15) which is associated with Usher syndrome type 1F when mutated. Zebrafish carry two copies of this gene - pcdh15a and b - which have previously been documented as having spatial separation: pcdh15a appears in the inner ear and neuromasts, while pcdh15b is exclusively seen in photoreceptors of the retina. Inactivation of pcdh15b has been shown in the past to be a model of human Usher 1F, though the phenotype is less strong than that seen in human patients.

In this manuscript, the authors study both single and double mutants of zebrafish pcdh15, and find that previous literature did not fully appreciate the redundancy of pcdh15 in the fish (and, they observe, this is a cautionary tale that applies to many gene duplicates in this species). Double pcdh15a/b mutants showed much more severe retinal phenotypes resembling human patients, as well as ear phenotypes. The experiments carried out use both structural histological analysis and functional visual testing (optokinetic response) to verify the full phenotype seen in both single and double mutants. Statistical tests are appropriate and well-powered.

Overall, this is a well-designed and executed study, which details the full spectrum of phenotypic effects seen in the zebrafish following inactivation of pcdh15a and b in a type of quasi-allelic series. My only quibble is in the language that is used to highlight the need to inactivate both copies of *every* gene that has been duplicated: I fully agree that this is the case with pcdh15 and likely many others. However, I would prefer to see the authors use language that communicates probability rather than absolutes - "it is likely that gene duplicates can compensate for one another, or become activated when one but not the other is inactivated". There may well be cases where compensation between paralogs does not happen. This is a minor point, and the manuscript is excellent overall.

Reviewer #3

(Remarks to the Author)
Thank you for the opportunity to review this manuscript entitled "Overlapping expression and co-operative function of the zebrafish pcdh15 paralogs-the case for generating double mutants when modelling human disease" by Chrystal et al. This is a well-crafted, well-drafted, rigorous set of experiments that takes up where Seiler et al left off in 2005 to use modern techniques to delineate a number of attributes of pcdh15a and pcdh15b.

This paper represents an important contribution and provides a model that be used to develop novel therapies for Usher Syndrome Type 1F, a human condition characterized by severe-profound deafness at birth with balance deficits followed by retinitis pigmentosa that can start as early as the first decade in patients with loss of function variants.

The title is much narrower than it really should be for the contributions of this manuscript as the authors provide a model that will have important implications for development of therapy and an understanding of the biology of PDCH15.

Important contributions:

1) The zebrafish retina more closely resembles the human retina than murine models and will serve as an important model for future work to understand biology of pcdh15 as well as to develop novel therapies.

2) That pcdh15a and pcdh15b work in a paralog and isoform specific manner and that double germline mutants of both paralogs results in a more severe inner ear and retinal phenotype than the single mutants alone. It was believed that each paralog functioned separately; pcdh15a was limited to neuromasts and inner ear hair cells and pcdh15b to photoreceptors. The investigators provide strong evidence on both hair cell and in the retina that both are necessary for cell function, morphology, as well as behavioral aspects of vision and hearing.

3) That zebrafish homozygous mutants, if raised separately, can survive long term when they are not forced to compete with wildtype and heterozygous tank mates. This is very helpful for the field in general and provides new strategies to study homozygous deaf circler mutants.

4) The rigor of the qRT-PCR experiments was appreciated, particularly with greater than 6 technical replicates and the use of multiple control transcripts.

There are two figure comments:

1) Figure S5, B: Are the figure legends reversed? The open space in the vertical bar graph represents the severe phenotype in 80% but the written legend reports that a mild phenotype is reported in fewer than 20%.

2) Mobility assays: Figure 6 and S3: The assays measure mobility with a light stimulus, yet these fish have impaired mobility and reduced swim bladder inflation, and this should be mentioned in the figure legend. The OKR assay mitigated this well and it is discussed in the text.

Reviewer #4

(Remarks to the Author)
I co-reviewed this manuscript with one of the reviewers who provided the listed reports. This is part of the Communications Biology initiative to facilitate training in peer review and to provide appropriate recognition for Early Career Researchers who co-review manuscripts.

Version 1:

Reviewer comments:

Reviewer #1

(Remarks to the Author)
The science in the original and revised manuscript is outstanding, rigorous and important. The authors responded appropriately to most of the suggested changes and preferred not to make other edits recommended, but they fall under the category of artistic license.

Reviewer #3

(Remarks to the Author)
The authors were responsive to the reviews.

Open Access This Peer Review File is licensed under a Creative Commons Attribution 4.0 International License, which permits use, sharing, adaptation, distribution and reproduction in any medium or format, as long as you give appropriate credit to the original author(s) and the source, provide a link to the Creative Commons license, and indicate if changes were made.
In cases where reviewers are anonymous, credit should be given to 'Anonymous Referee' and the source.
The images or other third party material in this Peer Review File are included in the article's Creative Commons license, unless indicated otherwise in a credit line to the material. If material is not included in the article's Creative Commons license and your intended use is not permitted by statutory regulation or exceeds the permitted use, you will need to obtain permission directly from the copyright holder.
To view a copy of this license, visit https://creativecommons.org/licenses/by/4.0/

Referee expertise:

Referee #1: Zebrafish biology

Referee #2: Cell Biology, Neurobiology and Anatomy

Referee #3: Hearing loss

Reviewers' comments:

Reviewer #1 (Remarks to the Author):

The manuscript from Chrystal and co-authors entitled “Overlapping expression and co-operative function of the zebrafish pcdh15 paralogs-the case for generating doble mutants when modelling human disease” is very nicely crafted. The authors provide scholarly science to clarify the function of the two pch15 paralogs in zebrafish and appropriately and directly correct long-standing errors in the literature about the claim of distinctly different nonoverlapping functions of pcdh15a and pcdh15b paralogs reported by prominent scientists. This study is robust important science that corrects the literature and adds considerable new data about the functions of pcdh15 paralogs in zebrafish.

There are no major concerns detected in the manuscript such as absence of important control data or essential missing data. There are remaining questions for future studies. However, there are several recommendations listed below for improving the manuscript starting with the title.

Thank you for your reviews of our manuscript, and for your kind words! We will address each of your recommendations in turn.

1. The title of the manuscript is too long. Consider “Overlapping expression and co-operative function of the zebrafish pcdh15 paralogs”. The case for studying double mutants has been obvious starting with studies of Drosophila melanogaster a century ago. The point is certainly worth emphasizing again in the abstract and the body of manuscript.

The manuscript is trying to explore two concepts concurrently. Firstly, we found that *pcdh15* paralogs have far more cooperativity than has been appreciated for the last two decades – and want to explicitly state this conclusion. Secondly, we feel that the data has much greater scope than just being a study of two paralogs. In crafting this manuscript, we hope to encourage other zebrafish researchers to reassess long-standing assumptions about subfunctionalization of related genes unless the evidence provided is substantial. Reviewer #2 suggests that this may be a cautionary tale for some, and we align with that view. As such, we have decided to retain the original title to ensure that both points are reflected from the outset.

2. Abstract, line 15, consider adding a phrase describing what is meant by “Transcriptional adaptation”. If there is insufficient room in the abstract, define TA the first time used rather than waiting for a definition of TA in the body of the manuscript on line 136.

Thank you for this suggestion. We have now included a brief description of transcriptional adaptation in the introduction section of the manuscript: line 50, starting “However, the retention of paralogous genes…”

3. Introduction, line 59, Name the three CD1, CD2 and CD3 at the end of this sentence when first mentioned and provide a reference for the three cytoplasmic domains when CD1, CD2 and CD3 were first reported in mouse.

To line 61 we have added parentheses naming the CD1-3 isoforms: "(C-terminal cytoplasmic domain 1-3)". We have also added a reference to the original paper that first reported on the alternate usage of the C-terminal domain: The Tip-Link Antigen, a Protein Associated with the Transduction Complex of Sensory Hair Cells, Is Protocadherin-15, 2006, Ahmed et al.

4. The authors are correct that Pcdh15 mouse models fail to recapitulate the more severe human RP phenotype resulting in progressive loss of vision. However, careful examination of vision physiology of a Pcdh15 mouse model does have abnormalities although not nearly as devesatating as in human with the same truncating variant of PCDH15 (Sethna et al 2021, eLife).

We agree with this comment and feel that it has been adequately addressed in the discussion section, line 410: "Murine models of USH1 do possess mild visual deficits, but do not have pronounced histological changes or retinal dystrophy[35,36,72-77]". As requested, we have included a citation to the Sethna eLife paper here.

The subtle retinal phenotypes observed in murine models are out of the scope of this study, but we do want to contrast the lack of retinal cell death in mouse models to our novel zebrafish mutant.

5. Introduction, line85, "dichotomic expression pattern" is pedantic phraseology?

We believe that this is a concise and precise way to communicate the previous model of pcdh15 subfunctionalization.

6. Results, line 202, "bathing" for how long?

Apologies if the methods section was unclear. We have modified line 561 of the FM 1-43 uptake methodology to match the description in the results: "bathed in FM1-43FX working solution for 1 minute…"

7. Line 289, what criterion was used to claim nuclei are pyknotic?

Apologies for lack of clarity. I have added details to the methods section under "bright light rearing" for clarity. See line 571 starting "Pyknotic nuclei were identified…"

8. Lines 301 to 322. There are several terms used without criteria stated including "displaced", "disordered", "irregular", and "disorganized" that would be better science if vague words were associated with some specific detailed data or quantification mentioned in the text.

Where possible, we have attempted to associate altered phenotypes in the mutants with specific measurements describing these changes, such as number and length of outer segments when describing this phenotype as irregular and disordered. In other cases where the phenotype is overtly distinct from the WT, such as lack of parallel arranged CPs, we described these as disorganized without quantification as this was an obvious deviation from the typical arrangement.

9. Figure 8 Panel F and G, consider including plots comparing UV OS quantification and blue OS quantification as shown in figure 2 panel D and F. This would strengthen statements on effect of mixed zygosity of pcdh15.

Thank you for your suggestion. We have now included an additional supplemental figure (Fig. S5) which quantifies the difference in number and length of the respective outer segments in mixed zygosity mutants versus age-matched WT controls.

10. Line 319, consider moving the sentence on rod cell susceptibility earlier in the Results section where pcdh15b-/- showing mild outer segment dysmorphology is discussed.

Thank you for your suggestion. We have tried to keep the order of the phenotypic assessments uniform throughout the manuscript. We have therefore kept the rod assessment data at the end of the section to mirror assessment of the pcdh15b-/- single mutant (line 125 onwards and Fig. 2G, H).

11. Consider moving the section beginning at line 323 titled "Isoform-specific roles for pcdh15" to the beginning of the Results section. The Results section would end with a better transition to the Discussion section with the last sentence of the Results "Taken together, these data reveal…..".

Thank you for your suggestion. We can see how rearranging the results in this way could improve the flow of the manuscript – by grouping all the studies on individual transcripts together. However, the disadvantage would be that we would jump from discussing stable zebrafish lines (Fig. 2, 3A), to discussing crispant models (Fig. 9, S5), and then back to stable lines. We think that both approaches have merit, but to avoid confusion about what model we are using, we have decided to retain the original order of the experiments.

12. Discussion, As mentioned by the authors, on one side of a tip link are two protocadhein 15 monomers in handshake with two monomers of cadherin 23 as shown in panel B of figure 1. Not mentioned is that during development and when tip links are broken, protocadherin 15 forms tip links on both side without cadherin23. Subsequently, cadherin 23 replaces protocadherin on the upside of the tip link. See Indzhykulian et al., PLOS Biology. For the future, is this phenomenon reported for mouse present in zebra fish?

This is an excellent point about protocadherin related 15 having the capacity to form both sides of the handshake interaction between adjacent stereocilia and would justify future investigation to determine conservation in the zebrafish. However, (despite trying) we have not found a commercial antibody that cross-reacts with zebrafish cadherin related 23 proteins so we are unable to attempt this experiment.

13. Materials and Methods, line 455, add details of the fixation not just 4% paraformaldehyde.

Apologies, we have added more detail to the fixation protocol on line 445.

14. Is it Spurr epoxy resin not Spurr's?

Thank you, we have made this correction on line 497.

15. Most of the figures have a font size that is too small even though there is plenty of room to increase the font size.

Noted. Once we receive proofs and can determine the actual size that our figures will be when in print, we will be happy to increase text size or modify figure layouts to conform with the journal's specifications.

16. Figure 1, panel A, the CD1, CD2 and CD3 are labelled "Cytoplasmic" but the key calls them C-terminal domain. Both are correct but consider using one or the other but not both in the same figure panel.

Thank you, we have now modified the figure and legend to include the full name: "C-terminal cytoplasmic domain" when first introduced. Subsequent discussion uses the contracted "C-terminal domain".

17. Figure 1 legend, "large gene" is meaningless and not very scientific. Provide some numbers either the number of exons and or the number of base pairs.

Agreed. We have changed the figure legend to "1.8 Mb gene".

18. Figure 1 panel A, in the table add similarity percents to identity percents.

Our apologies but we do not understand this suggestion. The table is depicting the number of residues where identity of the amino acid is conserved. As such we have not made any changes here.

19. Figure 1, panel B, the MET channel is on the side of a stereocilium not on the top as drawn.

Our understanding of the literature is that we still have an incomplete characterization of the MET channels, but that several protein subunits provide significant contribution e.g. LHFPL5, TMC1, TMC2, TMIE. The depiction in Figure 1 is inspired by other schematics from research groups that have studied MET channels in detail. Please refer to PMID: 36223766 Fig. 2B, and PMID: 40280017 Fig. 4. Also, given that EM data locates PCDH15 expression at the top of the stereocilium (PMID: 34964715 Fig. 4), and that PCDH15 interacts with the LHFPL5 subunit to gate the channel, it seems reasonable to infer that the complex is localized in this way.

20. Supplementary figure S1, panel B, does the heterozygote fail to inflate the swim bladder? Why is there a black arrow pointing to the swim bladder for heterozygotes?

We apologise for this error. A subset of homozygotes do fail to inflate the swim bladder, however this represents <30% of individuals. We therefore replaced the middle panel in Supp. Fig. 1B (6 dpf) to a heterozygote with swim bladder inflation to provide a more representative example of the phenotype.

21. Figure 2, legend, Indicate why the Welch t-test was used. Is this because of unequal variances?

Due to the low number of biological replicates (<30) inherent to the disease modelling data presented within this manuscript, the Welch t-test was favoured. The Welch t-test makes fewer assumptions about the data compared to a student t-test (i.e. equal variance between groups) and provides a more conservative estimation of statistical significance.

22. Figure 2 Panel D, F, H, consider including the count of samples "n" for consistency between other figures and include significance bars. Include counts for figures 5, 6, S3, S4, 7, 8 and 9.

Thank you for your suggestion. When all groups on a graph have equal n-values, we have included this in the figure. When n-value is different between conditions, we have added this to the legend. We will also be publishing all raw data, so interested parties will have access to this information.

23. Figure 9 Panel B, SB inflation coloring is confusing with inflated and not inflated, inconsistent with previous SB inflation shown in Supplementary Figure S1 Panel C. Also, n=3 representing clutches in this figure is unclear. Consider providing the number of fish instead.

The colouring used in Fig. 9B was the same, but the key was incorrect. Red = uninflated. White = inflated. We have corrected this error. We have also changed the title above the light micrographs in this panel from "CRISPR" to "crispant" to better describe what the panel is displaying.

24. Figure 9 Panel D shows splayed stereocilia and attached kinocilium, which the authors claim indicates a CD3 role in mechanosensation. Are there any comparisons that could be made to figure 5 panel F which shows pcdh15a-/- and pcdh15b-/- double mutant showing splayed stereocilia and detached kinocilium?

Thank you for this suggestion. To further explore these data, we have added a new paragraph to the discussion starting line 411, "In contrast to vestibular hair cells…" Kinocilial detachment can be attributed to pcdh15b (Fig. 5) butt is not observed when we generated ΔCD1 or ΔCD3 crispants. We have therefore speculated that connection of the kinocilium to stereocilia may be dependent upon pcdh15-CD2 isoforms, while acknowledging that we cannot discount involvement from multiple isoforms.

Reviewer #2 (Remarks to the Author):

Chrystal and colleagues present a very nice manuscript that studies the effects of inactivation of zebrafish genes which are duplicated in this species alone. In this case, they study orthologs of PCDH15 (protocadherin 15) which is associated with Usher syndrome type 1F when mutated. Zebrafish carry two copies of this gene - pcdh15a and b - which have previously been documented as having spatial separation: pcdh15a appears in the inner ear and neuromasts, while pcdh15b is exclusively seen in photoreceptors of the retina. Inactivation of pcdh15b has been shown in the past to be a model of human Usher 1F, though the phenotype is less strong than that seen in human patients.

In this manuscript, the authors study both single and double mutants of zebrafish pcdh15, and find that previous literature did not fully appreciate the redundancy of pcdh15 in the fish (and, they observe, this is a cautionary tale that applies to many gene duplicates in this species). Double pcdh15a/b mutants showed much more severe retinal phenotypes resembling human patients, as well as ear phenotypes. The experiments carried out use both structural histological analysis and functional visual testing (optokinetic response) to verify the full phenotype seen in both single and double mutants. Statistical tests are appropriate and well-powered.

Overall, this is a well-designed and executed study, which details the full spectrum of phenotypic effects seen in the zebrafish following inactivation of pcdh15a and b in a type of quasi-allelic series. My only quibble is in the language that is used to highlight the need to inactivate both copies of *every* gene that has been duplicated: I fully agree that this is the case with pcdh15 and likely many others. However, I would prefer to see the authors use language that communicates probability rather than absolutes - "it is likely that gene duplicates can compensate for one another, or become activated when one but not the other is inactivated". There may well be cases where compensation between paralogs does not happen. This is a minor point, and the manuscript is excellent overall.

Thank you for reviewing our manuscript and for your praise of our work! It has been a joy to read these reviewer comments and learn that our peers appreciate the significance of this study.

We agree that the language around genetic compensation was too strong and have modified paragraph 1 of the discussion to soften the message. This section now starts “highlight a possible confound for zebrafish models…” and begins on line 352.

Reviewer #3 (Remarks to the Author):

Thank you for the opportunity to review this manuscript entitled “Overlapping expression and co-operative function of the zebrafish pcdh15 paralogs-the case for generating double mutants when modelling human disease” by Chrystal et al. This is a well-crafted, well-drafted, rigorous set of experiments that takes up where Seiler et al left off in 2005 to use modern techniques to delineate a number of attributes of pcdh15a and pcdh15b.

This paper represents an important contribution and provides a model that be used to develop novel therapies for Usher Syndrome Type 1F, a human condition characterized by severe-profound deafness at birth with balance deficits followed by retinitis pigmentosa that can start as early as the first decade in patients with loss of function variants.

The title is much narrower than it really should be for the contributions of this manuscript as the authors provide a model that will have important implications for development of therapy and an understanding of the biology of PDCH15.

Important contributions:

1) The zebrafish retina more closely resembles the human retina than murine models and will serve as an important model for future work to understand biology of pcdh15 as well as to develop novel therapies.

2) That pcdh15a and pcdh15b work in a paralog and isoform specific manner and that double germline mutants of both paralogs results in a more severe inner ear and retinal phenotype than the single mutants alone. It was believed that each paralog functioned separately; pcdh15a was limited to neuromasts and inner ear hair cells and pcdh15b to photoreceptors. The investigators provide strong evidence on both hair cell and in the retina that both are necessary for cell function, morphology, as well as behavioral aspects of vision and hearing.

3) That zebrafish homozygous mutants, if raised separately, can survive long term when they are not forced to compete with wildtype and heterozygous tank mates. This is very helpful for the field in general and provides new strategies to study homozygous deaf circler mutants.

4) The rigor of the qRT-PCR experiments was appreciated, particularly with greater than 6 technical replicates and the use of multiple control transcripts.

Thank you for reviewing our manuscript, and for your generous appraisal of this work. We have addressed your comments below.

There are two figure comments:

1) Figure S5, B: Are the figure legends reversed? The open space in the vertical bar graph represents the severe phenotype in 80% but the written legend reports that a mild phenotype is reported in fewer than 20%.

Our apologies, the text in the key to the graph was reversed. Thank you for pointing this out. It has now been rectified.

2) Mobility assays: Figure 6 and S3: The assays measure mobility with a light stimulus, yet these fish have impaired mobility and reduced swim bladder inflation, and this should be mentioned in the figure legend. The OKR assay mitigated this well and it is discussed in the text.

Thank you for your suggestion. We have corrected this in the figure legend: "However, swimming distance was lower in all mutant groups regardless of light intensity (Fig. S3)"

Reviewer #4 (Remarks to the Author):

I co-reviewed this manuscript with one of the reviewers who provided the listed reports. This is part of the Communications Biology initiative to facilitate training in peer review and to provide appropriate recognition for Early Career Researchers who co-review manuscripts.

Thank you for your reviewing our manuscript, and we hope that the process was helpful for your development.

REVIEWERS' COMMENTS:

Reviewer #1 (Remarks to the Author):

The science in the original and revised manuscript is outstanding, rigorous and important. The authors responded appropriately to most of the suggested changes and preferred not to make other edits recommended, but they fall under the category of artistic license.

Thank you for your constructive reviews.

Reviewer #3 (Remarks to the Author):

The authors were responsive to the reviews.

Thank you for your constructive reviews.
