# Supplementary material for: Overlapping expression and co-operative function of the zebrafish pcdh15 paralogs
Source: Commun Biol. 2026 May 18;9:1048. doi: 10.1038/s42003-026-10289-7 (PMC13443151; doi:10.1038/s42003-026-10289-7)
Supplement: Supplementary file 4 [file 42003_2026_10289_MOESM2_ESM.docx]

**Description of Additional Supplementary Files**

File name: Supplementary Data

Description: Source Data behind the graphs in the paper

Supplementary Video 1 – pcdh15a-/-; pcdh15b-/- double homozygote larvae display uncoordinated swimming behaviour consistent with vestibular dysfunction.

Supplementary Video 2 – pcdh15a+/-; pcdh15b-/- mixed zygosity double mutants display uncoordinated swimming behaviours from ~15 mpf consistent with worsening equilibrioception and/or visual function.
